# Evaluation of antibacterial, antioxidant, and anti-inflammatory properties of GC/MS analysis of extracts of *Ajuga. integrifolia* Buch.-Ham. leaves

**DOI:** 10.1038/s41598-024-67133-3

**Published:** 2024-07-20

**Authors:** Harsha Singh, Suresh Kumar, Atul Arya

**Affiliations:** Medicinal Plant Research Laboratory, Department of Botany, Ramjas College, University of Delhi, New Delhi, India

**Keywords:** Drug discovery, Pharmacology

## Abstract

The purpose of the current study was to examine chemical composition, antioxidant, anti-inflammatory and antibacterial properties of leaves extract of *Ajuga integrifolia* Buch.-Ham. The antibacterial and antioxidant properties of three different solvents i.e. methanol (AIM), hexane (AIH), and water (AIW) were tested against two bacterial strains *Staphylococcus aureus* and *Escherichia coli*. The presence of antioxidant and antibacterial chemicals, such as hexanedioic acid, hexadecanoic acid, nonadecadiene, hexadecen-1-ol, octadecadienoic acid, nonane, phytol, henicosanal, stearyl aldehyde, and neophytadiene, were depicted in the GCMS chromatograms of three extracts. After the extracts' FTIR peaks were examined, it was discovered that phenols, amines, hydroxy groups, and components linked to amino acids were present. Compared to the Hexane and Water extracts, the Methanol extract showed a greater phenolic (196.16 ± 0.0083 mg gallic acid equivalent/gram), flavonoid (222.77 ± 0.002 mg rutin equivalents/g) and phosphomolybdate assay for total antioxidant activity (557.62 ± 0.0023 mg AAE/g). Methanol extract showed the highest scavenging activity with a minimum IC50 value was observed in DPPH assay. AIM showed its maximum anti-denaturation activity i.e. 3.75 ± 0.28%. For antibacterial activities, best zone of inhibition (ZOI) and minimum inhibitory concentration (MIC) was observed in case of the methanol extract as compared to other extracts against methicillin-resistant *Staphylococcus aureus* and β-lactam-resistant *Escherichia coli*.

## Introduction

Medicinal plants have been used widely in the food, cosmetics, medical, and other industries. Antibacterial and antioxidant levels in medicinal plants are very high^1^. As free radical scavengers, antioxidants help treat a variety of illnesses that produce reactive oxygen species (ROS) and harm cells by converting oxidants into their less harmful forms^2^. Because of their antioxidant properties medicinal plants are now being used as a natural component in herbal medicines as well as cosmetics^3^. Antioxidant compounds such as polyphenols can delay or inhibit the production of free radicals. Antioxidants are known as free radical scavengers. Free radicals are generally produced during respiration in the cell^4^. Both microvascular and macrovascular conditions, including cancer, asthma, diabetes, cardiovascular disease, stroke, infertility, neurological illnesses, and urolithiasis, are influenced by oxidative stress in their growth (Kain et al., 2020). When the body is under stress, it produces more reactive oxygen species (ROS and RNS) such as superoxide anion radicals, hydrogen peroxide, and hydroxyl radicals than it does non-enzymatic antioxidants like tocopherol (vitamin E), ascorbic acid, carotenoids, glutathione, and flavonoids and enzymatic antioxidants like superoxide dismutase (SOD), catalase, and glutathione peroxidase (GPx)^5^. The pharmaceutical business of today is moving towards historically utilised medicinal plants in an effort to lessen the cellular effects of synthetic medications. Plant contains numerous secondary metabolites such as terpenes, phenolics, and alkaloids etc. which have significant importance in pharmacology. *A. integrifolia* Buch.-Ham. (Syn: *Ajuga bracteosa*) is one of the most important herbaceous medicinal plant belonging to family lamiaceae which is found in the higher altitudes of Himalayan range including Himachal Pradesh, Uttarakhand, Jammu and Kashmir. A crude extract from the entire plant is used by the Indian tribal communities to treat fever, skin diseases, diabetes, asthma, and jaundice^6^. Bioactive chemicals found in *A. integrifolia* include phytoecdysones, favonol glycosides, ergosterol-5,8-endoperoxide, neo-clerodanediterpenoids, and iridoid glycosides with potent antioxidant and anti-inflammatory properties. *A. integrifolia* has been widely used as an antibacterial, anti-inflammatory, anti-arthritic, cardiotonic, antimalarial, anticancer, and antioxidant agent due to their potent components^7^. A study published recently described the ability of *A. bracteosa* nanoparticles to heal wounds^8^. *A. integrifoila* has antihypertensive properties, because of the flavonoid and iridoid glycoside content. Use of *A. integrifolia* for anti-cancer purposes as described in ethnobotanical and pharmacological studies^9^. The presence of secondary metabolites such as polyphenols, flavonoids shows significant antioxidant properties. While *A. integrifolia's* n-hexane extract demonstrated herbicidal action against *Lemna minor*, its methanolic extract was previously proven to be a powerful anticancer agent against MCF-7 and Hep-2 cell lines^10^. *A. integrifolia* extract demonstrated remarkable anti-plasmodial and anti-inflammatory effects in in vivo mouse models^11^. The higher wound-healing ability of A. bracteosa compared to Ajugarin I alone may be correlated with the antioxidant and antibacterial activities. Moreover, Ajugarin I appears to have a significant positive impact on A*. bracteosa's* wound healing characteristics^12^. Ahanger et al. successfully encapsulated ZIF-8 with *Ajuga parviflora* extract and investigated their antibacterial potential. A. parviflora Benth. possesses strong antidiabetic and antioxidant properties in addition to a multitude of phytoconstituents that are pharmacologically active. Consequently, this plant may be utilised as a natural source for the investigation of natural antioxidants and antidiabetic medications^14^. The anti-inflammatory properties of *A. integrifolia* micro-shoot culture extract may also be particularly useful in preventing acute respiratory distress syndrome by strong viral-induced inflammatory stimuli^15^. Such intriguing properties are due to the wide array of bioactive compounds like diterpenoids, iridoids, phytoecdysteroids and phenols. The studies on *Ajuga bracteosa* has verified its importance for the treatment of rheumatism and other inflammation disorders^16^. A wide variety of methanolic and chloroform extract activities from various *A. bracteosa* was reported both in vitro and in vivo. The plant's aerial parts were extracted using methanol, which showed encouraging antioxidant and analgesic, depressive, anticoagulant, and anti-inflammatory effects in vivo^17^.

## Results and discussion

### Plant extraction yield

**Figure 1:**
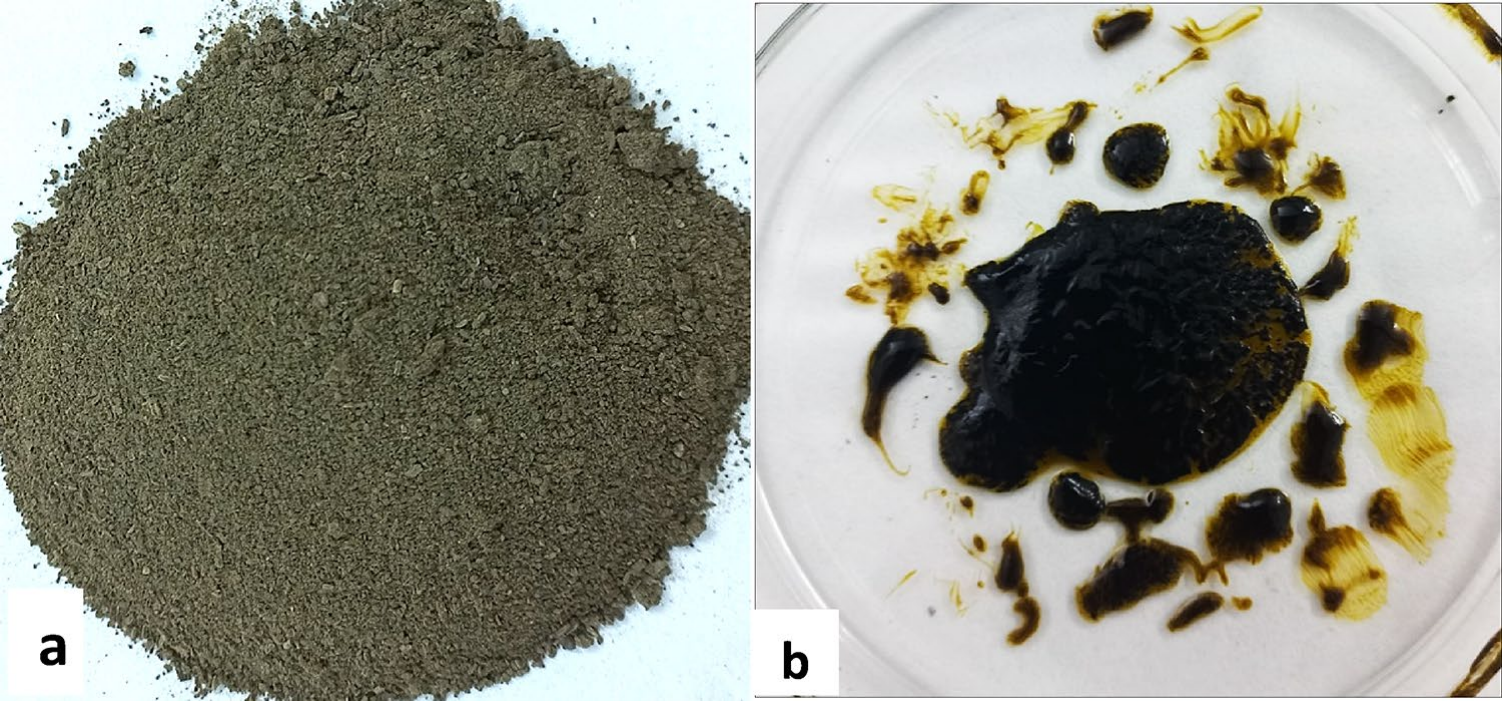
(**a**) Dried plant material. (**b**) Plant extract.

The extraction yield found in different solvent were as follow:

Methanol—3.98 g, Hexane—3.01 g, Aqueous—2.71 g (Fig. 1a,b).

The percentage extraction yield in different solvents (i.e. methanol, hexane and water) was calculated using formula

$${\text{Extractive Value }}\left( \% \right) = \left( {{\text{W1}} \times {1}00} \right)/{\text{W2}}$$

where W1 = weight (g) weight of plant powder after extraction, W2 = dry weight (g) of plant powder before extraction.

The percentage extractive value was found to be 15.92% in methanol, 12.04% in hexane and 10.84% in water.

### Metabolite profiling

The three plant extracts were subjected to phytochemical tests in order to identify anthroquinone, phenolic compounds, alkaloids, saponin, cardiac glycosides, flavanoids, tannin, terpenoids, resins, gums, and mucilage. The results of phytochemical screening is compiled in Table S1 (Supplementary file).

**Figure 2:**
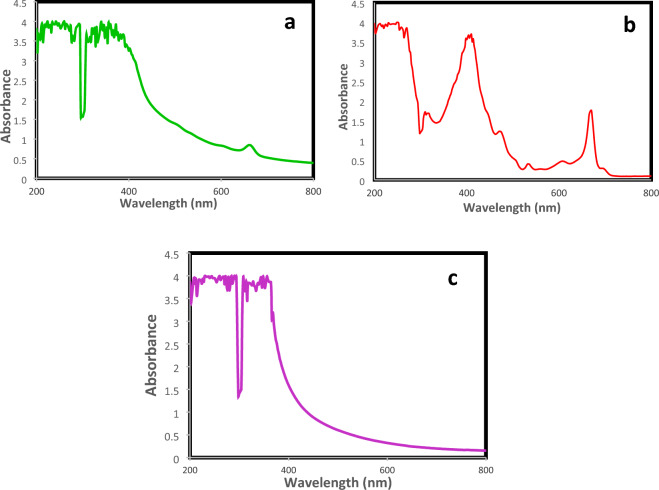
UV–visible spectrum analysis of *Ajuga integrifolia* extracts; (**a**) Methanol extract. (**b**) Hexane extract. (**c**) Water extract.

All three extracts' UV–Vis profiles showed a declining trend of absorption from 300 to 450 nm, which suggested the presence of tannins, carotenoids, alkaloids, phenolic chemicals, and flavonoids and their derivatives. The UV–Vis spectroscopy of methanolic extract represents a major peak of chlorophyll at 660 nm (Fig. 2a). The UV–Vis profile of hexane extract indicates the presence of three small peaks, i.e., 472, 538, 604 nm (Fig. 2b). confirms the presence of terpenoids and flavonoids; at 410 nm for alkaloids and phenols. A major peak at 668 nm confirms the presence of chlorophyll compounds. The aqueous extract represents the absorption spectra of water extract, which do not indicate any specific absorption peak from 400 to 800 nm and very smaller peaks in the range of 200–400 nm (Fig. 2c).

**Figure 3:**
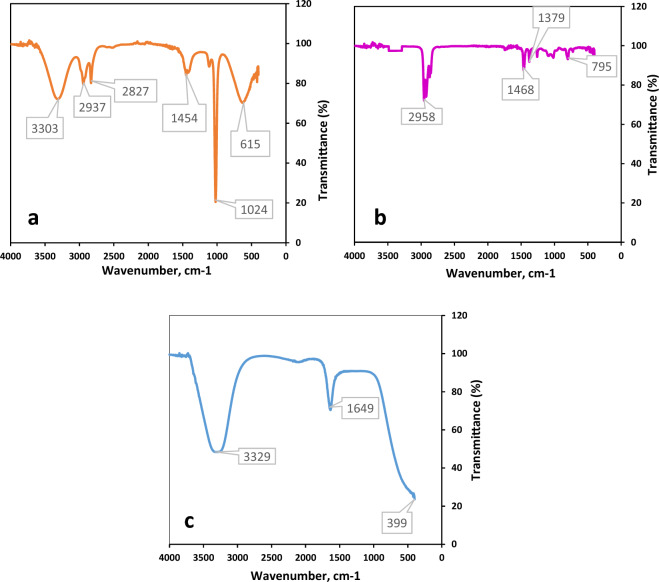
FTIR plots of *Ajuga integrifolia* extracts; (**a**) methanol extract. (**b**) Hexane extract. (**c**) Water extract.

The results of the FTIR plot, functional groups and their peak values are shown in Fig. 3. The FT-IR spectrum of extract showed a sharp peak at 1024 cm^−1^ which confirmed the presence of CN stretch of primary amine, methylene group and phosphate ions as well as show the presence of organic siloxane, primary amine, and aliphatic fluoro compounds. The sharp peaks at 3303 and 3329 cm^−1^ (Fig. 3a,c) designate imino compounds, and these peaks indicate the presence of = N–H stretch, organic nitrates and a secondary amine. Methylene C–H bend presence was supported by strong peaks at 1454 and 1468 cm^−1^. The C–H stretch stretch was observed at 2827 cm^−1^ and confirms the presence of methoxy compounds. The spectrum showed a peak at 2937, 2958 cm^−1^ which specifies the presence of Methyl C–H asym./sym. Stretch. The N–H and C=C stretches were observed at 1649 cm^−1^ and confirm the presence of aromatic ring stretch. The peak at 795 cm^−1^ showed the presence of C–H and C–O–O– stretch (Fig. 3b). The N–O stretch indicated the presence of nitrate ions at 1379 cm^−1^. The peak obtained at 399 cm^−1^ indicated the presence of N–H and O–H stretch. Peak at 615 cm^−1^ depicts the presence of C–O stretch and confirms the presence of thioether group.

A GC–MS chromatogram of methanol, hexane, and water extracts depicts the presence of 39, 44, and 53 bioactive compounds, respectively (Tables S2, S3, S4). The retention time, percentage area, nature of bioactive compounds, and biological activities are provided in Table S1. GCMS analysis of methanol extract shows the presence of numerous bioactive compounds (Table S2) along with their biological activities, such as Spiro[bicyclo[2.2.1]heptane-2,2'-[1,3]dioxolane]-3-one (22.31%). 3,5,5-Trimethylhexyl ethylphosphonofluoridate (21.76%). 1H-Naphtho[2,1-b]pyran-7-carboxylic acid, 3-ethenyldodecyl (14.56%), bruceantin (12.08%) possess strong anticancerous and antimalarial properties. Strong antibacterial properties have been reported for beta.-Phenoxyethyl methacrylate (8.06%), 2-thiabicyclo[3.1.0]hex-3-ene-3-carboxylic acid (3.85%), 6-Methyl-2-(tricos-14-en-1-yl)-2H-pyran-4(3H)-one (2.16%), propenoic acid (1.51%), 1-Hexadecanol (1.06%) and fatty acid methyl esters with varied percentage area. GCMS composition of hexane extract (Table S3) showed the presence of ledol (18.72%), Dodecane, 4,6-dimethyl (12.68%), 2,4-Hexanedione, 5-methyl-1-phenyl- (9%), Tetracontane (8.46%), Eicosane (6.9%), Decane, 3,7-dimethyl- (6.23%), glycidyl palmitate (4.25%), eicosane (4%), phytol (4.26%), Cholest-22-ene-21-ol, 3,5-dehydro-6-methoxy-, pivalate (4.22%), Pregn-5-en-3-ol, 20-methyl-21- (3.12%), Heptadecane (2.52%), Heneicosane (2.16%), 1-Acetoxypregnenolone (2.01%), Stigmasterol (1.66%), erythro-9,10-Dibromopentacosane (1.30%) and Octane, 5-ethyl-2-methyl- (1.17%).GCMS metabolite profiling of water extract (Table S4) indicates the presence of 2,4-Hexanedione, 5-methyl-1-phenyl- (11.72%), 4H-Pyran-4-one, 2,3-dihydro-3,5-dihydroxy-6-methyl (10.31%), ,3,4,5-Tetrahydroxy-cyclohexanecarboxylic acid (6.92%), Spiro[bicyclo[2.2.1]heptane-2,2'-[1,3]dioxolan]-3-one, 1,7,7-trimethyl- (6.50%), Acetamide, N-(4-oxo-1,4-dihydro-2H-quinazolin-3-yl) (4.98%), Hydrazine, 1,1-dimethyl (3.98%), Benzeneacetic acid, 2,5-dihydroxy (3.89%), Hydroquinone (3.65%), 1H-Naphtho[2,1-b]pyran-7-carboxylic acid, 3-ethenyldodecahydro-3,4a,7,10a-tetramethyl-, methyl ester (3.39%), 2-Cyclopenten-1-one, 2-hydroxy (3.30%), glycerin (3.25%), 2-Methoxy-4-vinylphenol (2.68%), 3,5-Dimethylphenyl isocyanate (1.96%), beta.-Phenoxyethyl methacrylate (1.78%), Cyclohexanecarboxylic acid, 3-methylene (1.65%), Benzenepropanoic acid (1.58%), Hexadecanoic acid, methyl ester (1.53%), 2-(2,3-Dimethyl-2-oxiranyl)pyridine (1.53%), 2,5-Anhydro-1,6-dideoxyhexo-3,4-DIULOSE (1.51%), 2,4-Dihydroxy-2,5-dimethyl-3(2H)-furan-3-one (1.45%), 2,3-Dihydro-benzofuran (1.43%), 2-Acetyl-2-hydroxy-.gamma.-butyrolactone (1.42%), Pentanal (1.27%), 2(4H)-Benzofuranone, 5,6,7,7a-tetrahydro-6-hydroxy-4,4,7a-trimethyl (1.13%), 2-Hydroxy-gamma-butyrolactone (1.05%), 2(3H)-Furanone, dihydro (1.04%). The evidence for fatty acid methyl esters as a potent antioxidant and antimicrobial agents has been provided earlier by Davoodbasha et al. which is also present in the methanolic and hexane extract of *Ajuga integrifolia.* Our GCMS shows few similarities with reports of Mothana et al. on *Ajuga bracteosa* extract that had 9,12-Octadecadienoic acid (1.22%), Docosanoic acid (0.62%), Hexadecanoic acid (1.13%) and possess antibacterial properties. According to Azemi et al.^18^, the bioactive compound neophytadiene (6%) is well-known for preventing bacterial problems, headaches, rheumatism, skin diseases, and diabetes. As an antidiabetic, anti-inflammatory, hypocholesterolemic, and larvicidal drug, hexadecanoic acid (12%) is utilised^19^. Another GC–MS study on *Ajuga bracteosa* methanolic extract showed the presence of Bruceantin (6.51), Stigmasterol (5.64), Pregnenolone (3.40%) that are known for their Antioxidant, Anti-inflammatory, Anticancer property^13^. According to Ismail et al.^20^, phytol (3.3%) has antibacterial, antioxidant, and antidiabetic properties. The hexane extracts had the highest percentage of a Sesquiterpene, Ledol (18.72%) which has been reported for its Antioxidant, antimicrobial, anti-fungus activity and hepatoprotective activity^21^. *Ajuga integrifolia* extract contains bioactive compounds that are beneficial for a variety of medical issues and function as strong biochemical agents that effectively treat infections, oxidants, and diabetes without causing any negative side effects. Moreover, *Ajuga integrifolia* is a rich source of phytochemical substances that may be employed in the production of new medications.

### Antioxidant activity

#### Quantification of total phenolic and flavonoid contents

**Figure 4:**
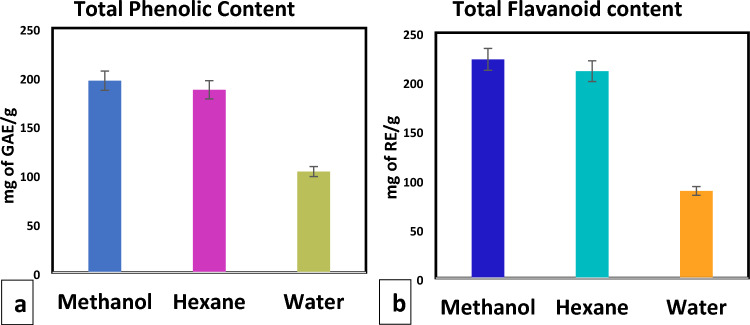
Quantification of (**a**) total phenolic and (**b**) flavonoid content in *Ajuga integrifolia* extracts.

In order to express the total phenolic contents of the *Ajuga integrifolia* extracts, the milligramme of gallic acid equivalents per gramme of plant extract was used. Using a calibration curve of gallic acid (y = 0.0007x + 0.1693, R^2^ = 0.9722), the experimental data were obtained (Fig. 4a). The methanol extract exhibited the highest phenolic contents (196.16 ± 0.0083 mg GAE/g), surpassing both the hexane and water extracts (186.87 ± 0.0081 mg GAE/g and 103.16 ± 0.0048 mg GAE/g, respectively) as shown in Fig. 4a. The amount of rutin equivalents (mg) in each gramme of *Ajuga integrifolia* extract was used to express the total flavonoid content. A rutin calibration curve (y = 0.0001x + 0.048, R^2^ = 0.94) was used to observe the experiment outcomes. The methanolic extract had the highest flavonoid content (222.77 ± 0.002 mg RE/g) than the hexane and water extracts (210.77 ± 0.0008 mg RE/g and 88.77 ± 0.001 mg RE/g, respectively) as shown in Fig. 4b.

#### Phosphomolybdate assay

**Figure 5:**
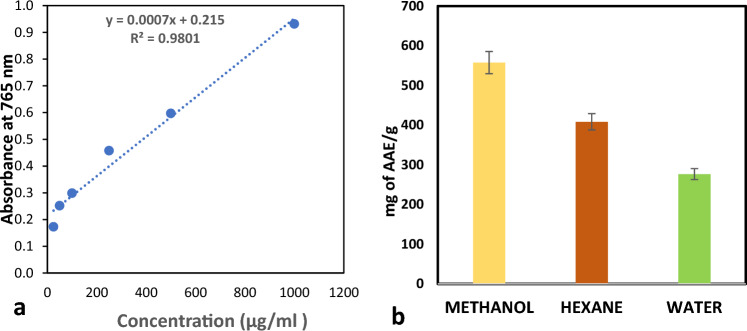
(**a**) Standard curve of ascorbic acid. (**b**) Antioxidant capacity of plant extracts.

The phosphomolybdate test was used to evaluate the overall antioxidant capability in extracts and ascorbic acid. The ascorbic acid equivalent (mg AAE/g) was utilised to generate the standard calibration curve (Fig. 5a). Total antioxidant capacity was influenced by drug dosage; that is, when we increased the plant extract concentration (25–1000 μg/ml), we saw a corresponding rise in scavenging capacity. The maximum radical scavenging activity was observed in the methanol extract at 1000 μg/ml (557.62 ± 0.0023 mg AAE/g), compared to hexane (408.57 ± 0.0011 mg AAE/g) and water extract (276.67 ± 0.0002 mg AAE/g) as depicted in Fig. 5b. The well-known secondary metabolites serve as the main antibacterial and antioxidant agents. The most abundant and varied class of natural antioxidants are phenols and flavonoids, which have a range of radial scavenging and bactericidal properties against pathogenic microorganisms.

#### DPPH free radical scavenging activity

**Figure 6:**
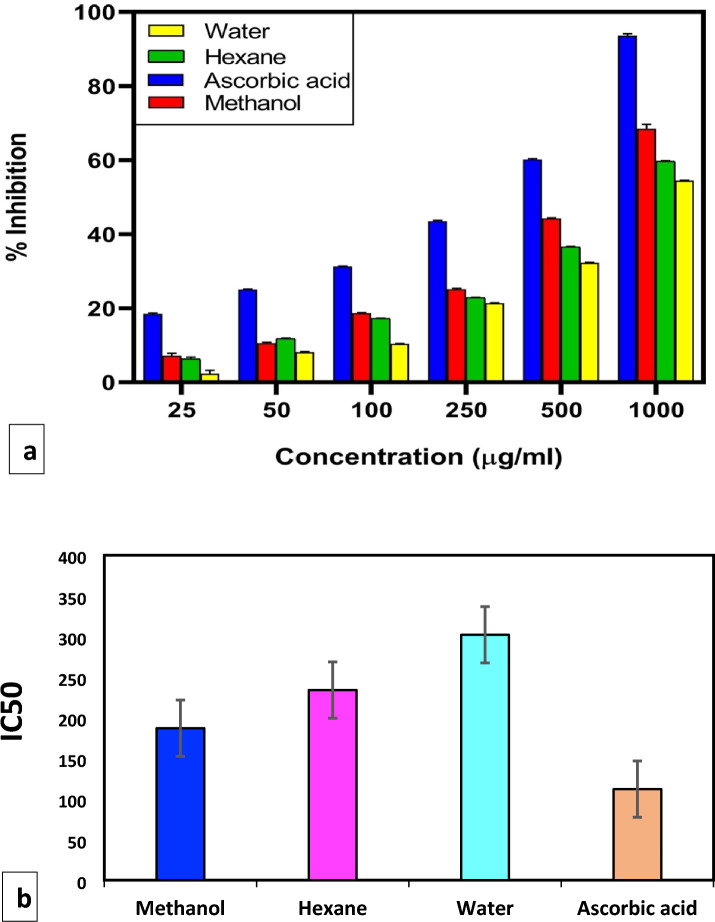
(**a**) Percentage inhibition of DPPH free radicals using *Ajuga integrifolia* extracts (methanol, hexane and water). (**b**) IC 50 values of plant extracts against DPPH radical performed using GraphPad Prism 8.

DPPH Assay on plant extracts exhibited significant scavenging activity in the current investigation in a dose-dependent manner (25–1000 μg/ml). Three solvent systems (methanol, hexane, and water) were contrasted with ascorbic acid. The rising concentration of extracts has a direct correlation with the scavenging activity. At 1000 μg/ml, methanol extract had the highest maximum DPPH radical scavenging activity (68.45 ± 1.24%) (Fig. 6a), followed by hexane and water extract (59.69 ± 0.16% and 54.48 ± 0.12%). Compared to hexane and water, the methanolic extract consistently had stronger antioxidant capabilities. Methanol extract had the highest DPPH radical scavenging potency because of its lowest IC50 value of 187 μg/mL, followed by Hexane and water (234 and 302 μg/mL, respectively) (Fig. 6b). The ascorbic acid employed as a standard and its IC50 value (112 μg/mL) and percentage scavenging activity at 1000 μg/ml (93.56 ± 0.603%) was found to be the lowest than all the three extracts. This study demonstrates that *Ajuga integrifolia* polar extract exhibits more antioxidant activity than its non-polar counterpart. According to Haminiuk et al. (2014), phenolic compounds exhibit higher polarity and greater solubility in polar settings, which could account for their potential.

### Anti-inflammatory activity

**Figure 7:**
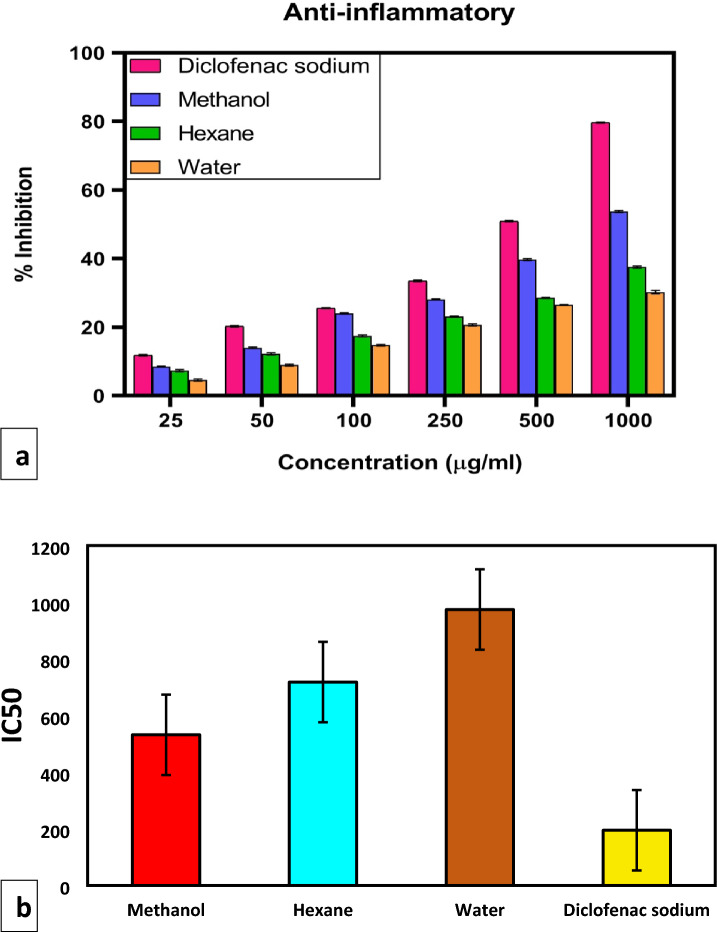
(**a**) BSA protein denaturation inhibition by *Ajuga integrifolia* extracts (methanol, hexane and water). (**b**) IC 50 values of plant extracts performed using GraphPad Prism 8.

In vitro BSA denaturation was used to test the anti-inflammatory properties of *Ajuga integrifolia* extracts. Using diclofenac sodium as the standard, concentration-dependent inhibition of BSA 25–1000 μg/ml) denaturation was seen with all three extracts. The anti-denaturation activity of methanolic extract at 1000 μg/ml was 53.75 ± 0.28%, followed by hexane and water i.e. 37.55 ± 0.32% and 30.21 ± 0.46% respectively. However, *Ajuga integrifolia* extracts were less effective than the diclofenac sodium with anti-inflammatory activity of 79.65 ± 0.18% (Fig. 7a). Saponins and tannins possess potent anti-inflammatory activity. The IC50 value for standard was least i.e. 195 μg/ml. However, the methanol extract has IC50 of 532 μg/ml followed by hexane and water (718 μg/ml and 974 μg/ml) as shown in Fig. 7b. In this study, we found that methanolic extracts were enriched with phenols, saponins and tannins. Therefore, it is responsible for inhibiting the denaturation of proteins to prevent the synthesis of autoantigens. 4-Hydroxy-3-methylacetophenone (0.16%) and 2-isopropyl-5-methylphenol (0.15%) present in the methanolic extract contributes to its anti-inflammatory properties^22^. However, presence of Heptadecane (2.52%) and Neophytadiene (0.77%) are also responsible for anti-denaturation activity in hexane extract^23^.

### Antibacterial activity

**Figure 8:**
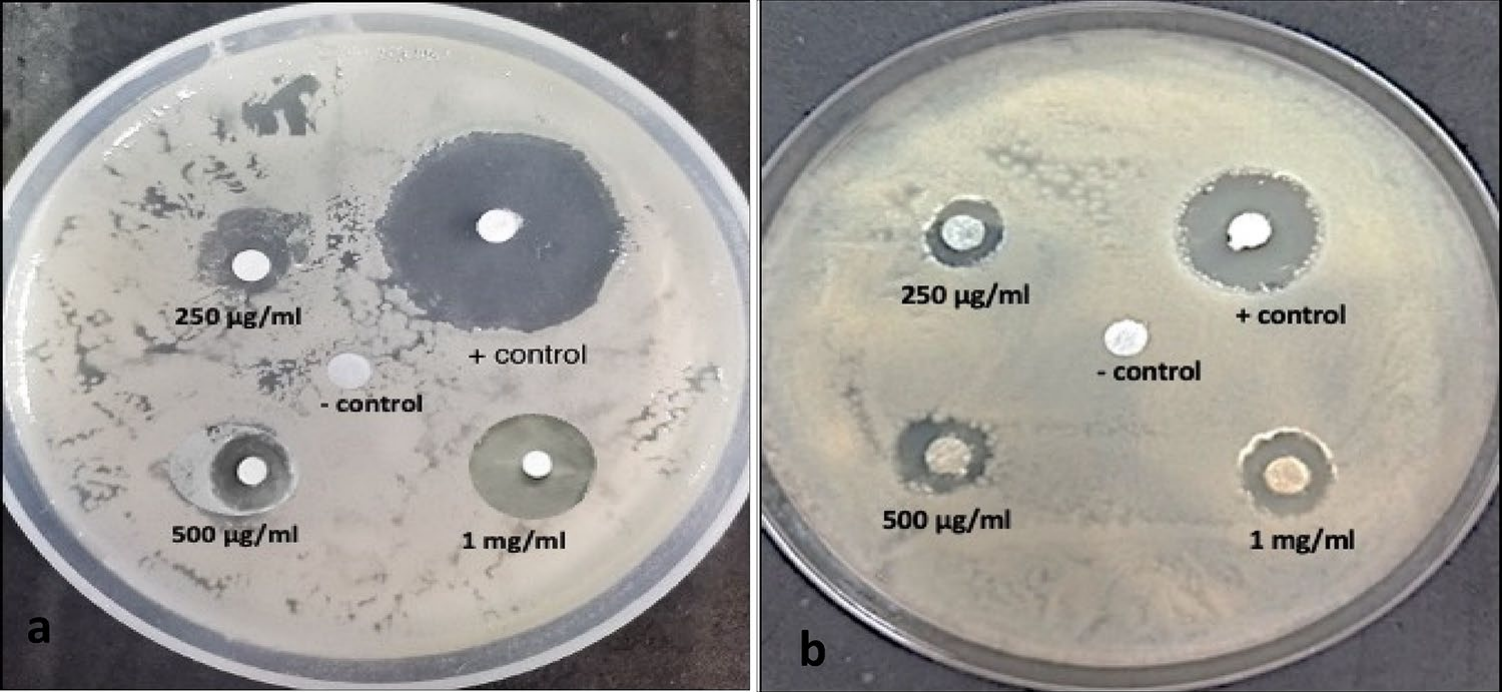
(**a**) *Staphylococcus aureus.* (**b**) *Escherichia coli.*

**Figure 9:**
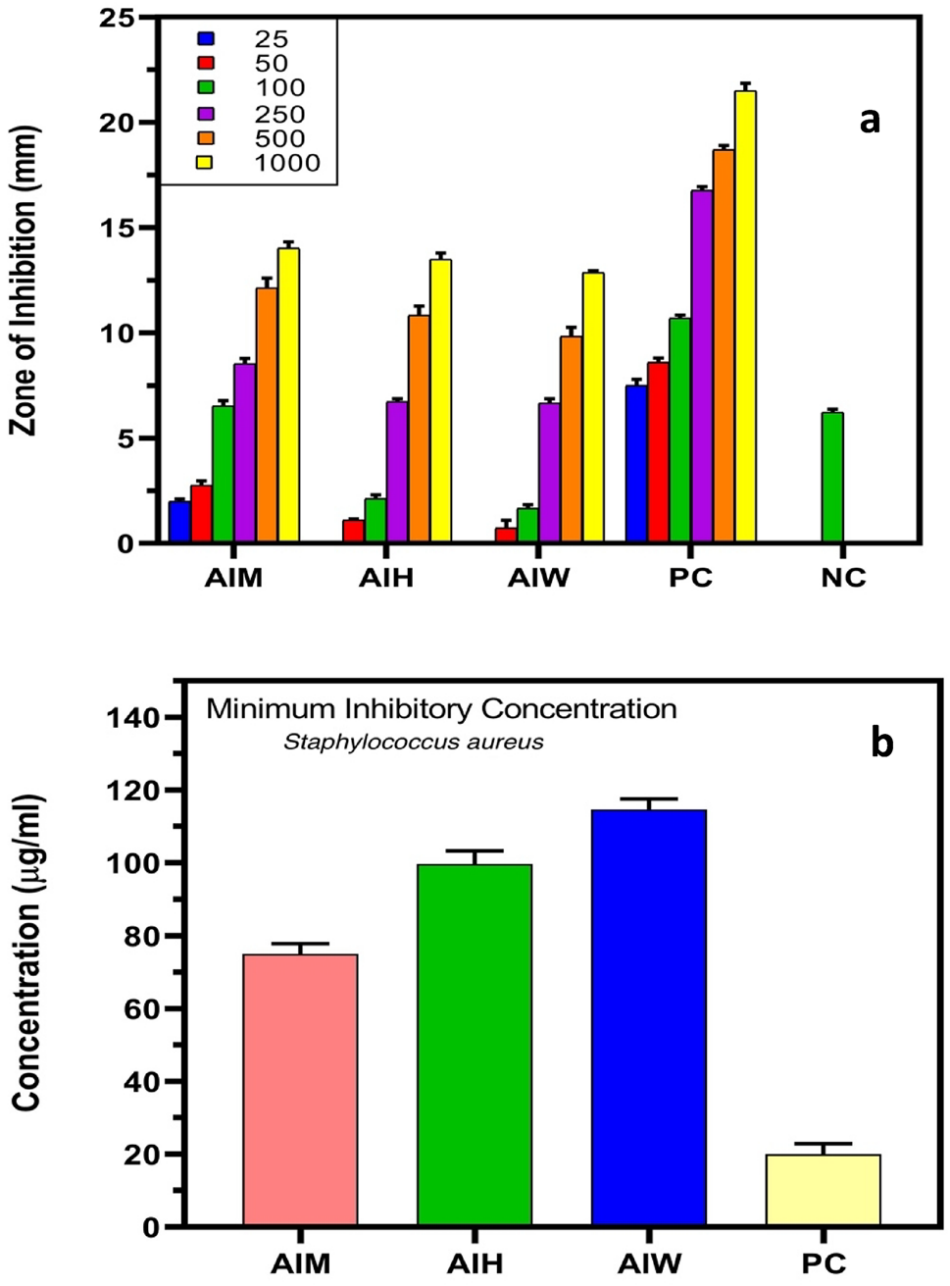
Antibacterial potential of *Ajuga integrifolia* extracts, i.e., methanol (AIM), hexane (AIH) and water (AIW) extracts. (**a**) Zone of inhibition (ZOI), (**b**) minimum inhibitory concentration (MIC) against *S. aureus.*

The antibacterial activity of AIM, AIH, and AIW extracts as well as gentamycin (positive control) against Gram-positive strain *Staphylococcus aureus* and Gram-negative bacterial strains of *Escherichia coli* was assessed using a disc diffusion assay as seen in Fig. 8a,b, the diameter of the bacterial inhibition zone in millilitres was evaluated in triplicate using varying concentrations of extracts (25–1000 (μg/ml), gentamycin (25–1000 (μg/ml), and methanol (99%) serving as a negative control. The minimum inhibitory concentration (MIC) and the zones of inhibition (ZOI) were each measured in triplicate. At different doses, gentamycin has the highest ZOI against both bacteria, followed by methanol, hexane and water. Graph pad prism 8.0 was used to evaluate the mean zone of inhibition (ZOI) for each experiment. The results are shown as the mean ± SEM of three replicates. At the initial two concentrations of 25 μg/ml and 50 μg/ml of plant extracts (methanol, hexane, and water), the diameter of inhibition zone was very less significant for the antibacterial activity. However, at comparable concentrations, gentamycin exhibited antibacterial activity against both bacteria. *Staphylococcus aureus* showed an increasing trend in ZOI from 2 to 14.33 mm, when the concentration of methanol extract was raised from 25 to 1000 μg/ml. According to Fig. 9a, AIH and AIW similarly showed increasing trends in ZOI, measuring 2.13 mm for AIH at 100 μg/ml to 13.5 mm at 1000 μg/ml and 1.66 mm at 100 μg/ml to 12.86 mm for AIW.

**Figure 10:**
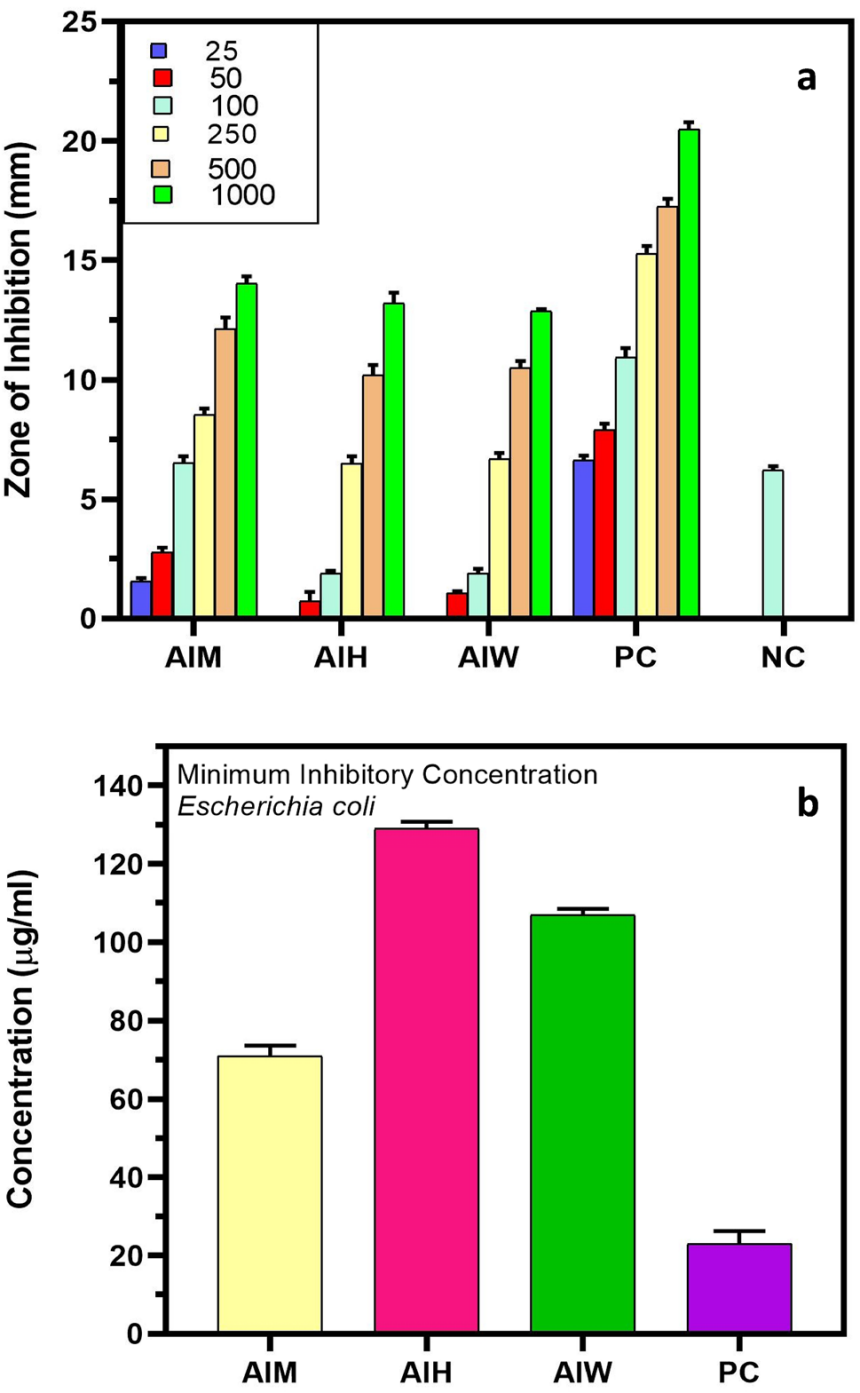
Antibacterial potential of *Ajuga integrifolia* extracts, i.e., methanol (AIM), hexane (AIH) and water (AIW) extracts. (**a**) Zone of inhibition (ZOI), (**b**) minimum inhibitory concentration (MIC) against *E.coli.*

Different antibacterial effects were demonstrated by AIM, AIH, AIW, and gentamicin against the two bacterial strains, with MIC values of 75, 99.6, 114.6, and 20 μg/ml for *Staphylococcus aureus* (Fig. 9b) and 71, 129, 107, and 23 μg/ml for *Escherichia coli* (Fig. 10b). As seen in Fig. 9a, AIM had superior antibacterial potency than hexane and water extract. The antibacterial activity of the alcoholic extracts was higher than that of the water and hexane extracts. Moreover, it has been shown that terpenes, fatty acid esters, phenols and their derivatives, and other chemicals found in alcoholic extracts can influence a number of target sites in bacterial cells^24^. Methanol, hexane, and water were utilised as control solvents, while gentamicin was utilised as the standard medication for the positive control.

The ZOI of *Escherichia coli* exhibited gradual increase for methanolic extract, ranging from 1.4 mm at 250 μg/ml to 14.03 mm at 1000 μg/ml. As illustrated in Fig. 10a, AIH and AIW likewise had rising trends in ZOI, with AIH rising from 1.9 mm at 100 μg/ml to 13.2 mm at 1000 μg/ml and AIW rising from 1.9 mm at 100 μg/ml to 12.16 mm at 1000 μg/ml. In every concentration, the standard drug gentamycin had a higher ZOI (8.6 mm at 25 μg/ml to 21.5 mm at 1000 μg/ml) than all the three extracts. Compared to gram-negative bacteria, gram-positive bacteria are more susceptible to the bioactive substances found in plant extracts. In addition to being costly and ineffectual in curing illnesses, synthetic drugs are frequently interfered with unfavourable side effects. Proteins make up the cell wall of bacteria. According to Kaczmarek^25^ tannins have the ability to attach to proline-rich proteins and obstruct protein synthesis. Proteins and enzymes are able to escape from cells due to the antibacterial activity of saponins^26^. Alkaloids have an antibacterial impact because of their capacity to limit cell division and interact with the DNA of both gram-positive and gram-negative bacteria. The list of bioactive substances with antimicrobial properties, including phytol, henicosanal, neophytadiene, and eicosanal, was also revealed by GCMS analysis. Microbial resistance is yet another unclear problem faced nowadays that can be dealt up by taking plants based medicines in daily lifestyle.

## Conclusion

It was observed that the *Ajuga integrifolia* extract had a large amount of bioactive components. The main justification for its therapeutic benefits is likely the synergistic impact of its bioactive components. The assessment of *Ajuga integrifolia* antioxidant and antibacterial components was reported using GCMS analysis. This study provides pharmacological evidence in favour of the traditional use of *Ajuga integrifolia* in the management and treatment of a number of human diseases and as an anti-inflammatory agent. Moreover, it has been declared that methanol extract is the most effective. According to our study, *Ajuga integrifolia* is a valuable natural source of antioxidants and can lessen the impact of generated ROS in the cell during specific illnesses. The study reports beneficial effects may be associated with the presence of significant bioactive metabolites, specifically the flavonoids, alkaloids, and phenols. This plant is a great substitute for anti-oxidant, anti-bacterial, and anti-diabetic medications, according to the results of the observed studies.

## Methods

### Chemicals

The following chemicals were purchased from Sigma-Aldrich and Merck: methanol, ethanol, LB agar, gentamicin, DPPH, sodium phosphate, sulfuric acid, ammonium molybdate, ascorbic acid, and NaCl. All of the chemicals were of good analytical grade (ACS grade).

### Collection and extraction

A freshly growing wild *Ajuga integrifolia* herb was manually collected from Nauni village (30° 49′ 26″ N 77° 10′ 44″ E), located in solan District of Himachal Pradesh, India. The collected plant material was dried, pressed, and pasted onto herbarium sheets. The specimen was identified and authenticated using The Plant List (theplantlist.org). The plant specimen was pressed and herbarium sheet was submitted for authentication at Raw Materials Herbarium and Museum, CSIR-NIScPR, New Delhi and an accession number (NIScPR/RHMD/Consult/2023/4576-68) was obtained for the voucher specimen. The collection was done on the guidelines which are issued by the National Medicinal Plant Board (NMPB), Ministry of Health and Family Welfare, Government of India. The dried plant sample was ground using an electric blender. Glass bottles were used to store the ground powder away from light, dust, and moisture until it was needed. After that, methanol, n-hexane, and water were used to selectively extract the powdered samples. Using a soxhlet extractor, 25 g of powdered leaves were extracted separately with 125 mL of methanol, hexane, and water at 55 °C, 50 °C, and 60 °C respectively for 48 h. After that, viscous semisolid masses were obtained by evaporating the solvent using a rotating vacuum evaporator. Prior to analysis, it was kept at 4 °C in dark containers.

### Plant extraction yield

The percentage extractive yield of whole plant extracts was calculated using the formula

$${\text{Extractive value }}\left( \% \right) = \left( {{\text{W1}}/{\text{W2}}} \right) \times {1}00$$

where W1 = weight (g) weight of plant powder after extraction, W2 = dry weight (g) of plant powder before extraction.

### Metabolite profiling

#### Phytochemical screening

To ascertain the presence and absence of phytochemicals such as alkaloids, cardio-glucosides, cardiac glycosides, flavonoids, phenolic compounds, tannin, saponin, terpenoids, anthroquinone, and phlobatannin, several extracts, including methanol, hexane, and aqueous, were examined (Table S1).

Ultraviolet (UV) was used to profile metabolites. We performed UV–visible spectroscopy in the 300–700 nm range using a DLAB UV-1000 spectrophotometer for the three extracts of *Ajuga integrifolia i.e*. Methanol (AIM), Hexane (AIH) and water (AIW). FTIR Spectroscopy with a frequency range of 4000–400 cm^−1^ was employed for the plant extracts, namely AIM, AIH, and AIW. For the GC–MS analysis of bioactive compounds, a Shimadzu QP2010 Plus with TD 20 thermal desorption system equipped with Omegawax 100 columns with ionisation of electrons and a constant flow rate of helium (1.21 mL/min) was utilised. The chromatogram's peak area was utilised to calculate the percentage of chemical components included in the extracts. The compounds were found by comparing their retention indices and using the libraries of the National Institute of Standards and Technology (NIST) and Wiley 8.

### Antioxidant activities

#### Total phenolic content

The method developed by Slinkard and Singleton^27^ was used to determine the total phenolic content (TPC) of the plant extract. 200 μl of Folin-Ciocalteu reagent, 1 ml ethanol, 3.16 ml distilled water, and 300 μl of plant extract (1 mg/ml) were added. After 8 min of incubation at room temperature, 600 μl of a 10% sodium carbonate solution was added. Test tubes were then covered with aluminium foil and incubated for 30 min at 40 °C in a water bath. Plant extract was substituted with an equal volume of ethanol for the blank. Using a UV visible spectrophotometer, the absorbance value was measured at 765 nm. Gallic acid is used to obtain the standard curve. The gallic acid equivalent (GAE) in milligrams (mg) per grams of extract was used to calculate the TPC.

#### Total flavonoid content

The technique of Ahmed et al.^28^ was used to calculate the total flavonoid content (TFC). 300 μl of extract (1 mg/ml), 3.4 ml of 30% aqueous ethanol, 150 μl of 0.5 M aq. sodium nitrite solution, and 150 μl of 0.3 M aluminium chloride solution were combined. After five minutes, one millilitre of sodium hydroxide solution was added, thoroughly agitated, and the absorbance at 506 nm was measured with a UV visible spectrophotometer. Plant extract was substituted with an equivalent volume of ethanol for the blank. Rutin was utilised to create a standard curve, and TFC was calculated as mg of rutin equivalent (RE) per g of extract.

#### Phosphomolybdate assay for total antioxidant capacity

The assay for phosphomolybdate, which was suggested by Khatoon et al.^29^, was used to estimate the total antioxidant capacity of the extracts. 3 ml of the phosphomolybdate reagent (28 mM sodium phosphate, 0.6 M sulfuric acid, and 4 mM ammonium molybdate) was added to 300 μl of plant extract (1 mg/ml). Test tubes were incubated at 95 °C for 90 min after being wrapped in aluminium foil. After allowing the reaction mixture to cool to room temperature, the absorbance at 765 nm was measured. Ascorbic acid was used to obtain the standard. Instead of using a plant sample, an identical volume of ethanol is utilised for the blank. TAC was calculated as ascorbic acid equivalents (AAE) milligrammes (mg) per gramme of extract.

#### 1,1‐diphenyl‐2‐picrylhydrazyl (DPPH) radical scavenging assay

The 1, 1-diphenyl-2-picrylhydrazyl (DPPH) free radical scavenging investigation was carried out using a method described by Brand-Williams et al.^30^ with minor modifications (Lalhminghlui and Jagetia^31^). By combining DPPH (2.4 mg) with ethanol (10 ml) and chilling the mixture, DPPH radical stock was obtained. To prepare the working solution for the DPPH radical, ethanol was added to the stock and diluted until the absorbance value at 517 nm was 0.98 ± 0.02. The plant extract (100 μl) in various concentrations was combined with 3 ml of DPPH working solution and allowed to sit at room temperature for 30 min in the dark. At 517 nm, the reaction mixture's absorbance was measured. The following formula was used to compute the inhibition percent:

$${\text{Percent inhibition}}: \, \left[ {\left( {{\text{A}}_{{{\text{control}}}} - {\text{A}}_{{{\text{sample}}}} } \right){\text{/A}}_{{{\text{control}}}} } \right)] \, \times { 1}00$$

where A_control_ and A_sample_ represent the absorbance of the DPPH solution and the DPPH solution containing standards or extracts, respectively.

### Evaluation of in vitro anti-inflammation activity

#### Inhibition of protein denaturation using Bovine serum albumin (BSA)

The anti-inflammatory activity of *Ajuga integrifolia* extracts (i.e. methanol, hexane and water) was determined in vitro to inhibit the denaturation of BSA according to methods described by Gunathilake et al.^32^ with some modifications. In this assay, the reaction mixture consisted of test extracts of *Ajuga integrifolia* and 0.5% BSA solution prepared in phosphate-buffered saline (pH 7.4). The pH of the reaction mixture was adjusted to 6.8 using a small amount of 1 N hydrochloride. The reaction mixtures were incubated at 72 °C for 5 min and allowed to cool. The absorbance of the control and test samples was determined using a spectrophotometer at 660 nm. Diclofenac sodium was used as standard. The experiment was conducted in triplicates.

$${\text{Percent inhibition}}: \, \left[ {\left( {{\text{A}}_{{{\text{control}}}} - {\text{A}}_{{{\text{sample}}}} } \right){\text{/A}}_{{{\text{control}}}} } \right)] \times {1}00$$

where A_control_ and A_sample_ represent the absorbance of the BSA solution and the BSA solution containing standards or extracts, respectively.

#### Antibacterial activity

Two bacterial cultures, i.e., Gram-positive *Staphylococcus aureus* (MTCC-11949) and gram negative bacteria *Escherichia coli* (MTCC-1652), were obtained from the Microbial Type Culture Collection (MTCC) located in Chandigarh, Punjab, India. The extract's antibacterial effectiveness was examined using a Kirby-Bauer single-disc susceptibility assay methodology. The pathogenic bacterial strains were raised in Luria–Bertani (LB) broth subculture medium for 12 h at 37 °C and 110 rpm with constant shaking. After that, the inoculum was collected by centrifuging for 10 min at 10,000 rpm. The positive and negative controls used were gentamycin and extract solvent, respectively. Sterilised discs (5 mm) dipped in varying extract concentrations (25–1000 μg/ml) were placed in the centre of the agar plate^33^. The antibacterial analysis was done by disc diffusion assay to check plant crude extract against mild pathogenic bacteria. Following that, parafilm was used to wrap agar petri plates, which were then kept at 37 °C for 10–16 h. Gentamycin was used as a positive control and solvent was used as a negative control. Zone of inhibition and minimum inhibitory concentration were calculated.

### Statistical analysis

Statistical analysis were performed from MS Excel 2019, GraphPad prism 8.0 and the Statistical Package for the Social Sciences (SPSS). Each experiment was conducted thrice (*n* = 3), and the results were expressed as the ± mean SEM (standard error of mean).

### Supplementary Information

Supplementary Tables.

## Data Availability

All the data are contained in this paper. Suresh Kumar, the corresponding author of this manuscript, will supply more information upon reasonable request. Email: suresh.kumar@ramjas.du.ac.in.
