# Supplementary material for: Evaluation of antibacterial, antioxidant, and anti-inflammatory properties of GC/MS analysis of extracts of Ajuga. integrifolia Buch.-Ham. leaves
Source: Sci Rep. 2024 Jul 20;14:16754. doi: 10.1038/s41598-024-67133-3 (PMC11271457; doi:10.1038/s41598-024-67133-3)
Supplement: Supplementary file 1 — Supplementary Tables. [file 41598_2024_67133_MOESM1_ESM.docx]

**Evaluation of antibacterial, antioxidant, and anti- inflammatory properties of GC/MS analysis of extracts of *Ajuga. integrifolia* Buch.-Ham. Leaves**

**Harsha Singh^1^, Suresh Kumar^1^*, Atul Arya^1^**

1. **Medicinal Plant Research Laboratory, Department of Botany, Ramjas College, University of Delhi**

**Corresponding Author: Suresh Kumar (**[**suresh.kumar@ramjas.du.ac.in**](mailto:suresh.kumar@ramjas.du.ac.in)**)**

**Supplementary information has five tables and references.**

Table 1: Phytochemical screening of extracts of *Ajuga. integrifolia*

| **S.No.** | **Name of test and Secondary metabolite** | **Methodology** | **Results** | **Methanol** | **Hexane** | **Water** | **References** |
| --- | --- | --- | --- | --- | --- | --- | --- |
| 1 | Wagner test Alkaloids | Add 2ml extract + 1% HCl + steam + 1ml of the solution with 6 drops of Wagner’s reagent | Brownish red Ppt | +++ | ++ | + | Chanda et al. 2006 |
| 2 | Kellar- Killiani test  Cardiac glycosides | 50 mg methanolic extract + 2 ml of chloroform + H_2_SO_4_ to form a layer. | Brown ring at interphase | ++ | + | - | Onwukaeme et al. 2007 |
| 3 | NaOH test  Flavanoids | Extract + dilute NaOH, + dilute HCl | Yellow solution on NaOH turns colorless on Hcl | ++ | + | + | Onwukaeme et al. 2007 |
| 4 | Lead acetate test  Phenolic compounds | To the test solution, a few drops of 10% lead acetate solution were added. | Formation of white precipitate | ++ | + | + | Bag and Singh 2013 |
| 5 | Frothing test  Saponin | 0.5ml extract + 5ml distilled water and shake well | Persistence of frothing | ++ | + | + | Parekh and Chanda 2007 |
| 6 | Braemer’s test  Tannin | 10% alcoholic FeCl_3_ + 2-3ml of methanolic extract (1:1) | Dark blue or greenish grey coloration | + | ++ | + | Parekh and Chanda 2007; Kumar et al. 2007 |
| 7 | Salkowski test  Terpenoids | 5ml extract + 2ml Chloroform + 3ml conc. H_2_SO_4_ | Reddish Brown color of interface | ++ | + | ++ | Edeoga et al. 2005 |
| 8 | Ammonia test  Anthroquinone | Add 1 ml of dilute (10 %) ammonia to 2 ml of chloroform extract. | A pink-red color in the ammoniacal (lower) layer | ++ | + | - | Onwukaeme et al. 2007 |
| 9 | HCL test  Phlobatannin | Extract was boiled with 2 ml of 1% hydrochloric acid. | Formation of red precipitate | + | - | + | Bag and Singh 2013 |
| 10 | Iodine test  Starch | The aqueous extract 5ml was treated with the reagent of the starch (iodine). | Blue color indicates the presence of starch | - | - | - | Zohra et al. 2012 |
| 11 | Gums and Mucilage  Alcohol Test | Dissolve 100mg extract in 10mL distilled water + 25mL  absolute alcohol (constant stirring) | White or cloudy precipitate | + | ++ | - | Whistler & BeMiller 1993 |
| 12 | Resins  Turbidity Test | 10mL extract + 20mL 4% HCl | Turbidity | - | - | - | Santhi and Sengottuvel 2016 |

Table 2: Chemical composition of methanol extract from *Ajuga integrifolia*

| **S. No.** | **Retention Time (min.)** | **Area%** | **Formula** | **Nature of Compound** | **Name of Compound** | **Retention Index** | **Biological Activity** | **Reference** |
| --- | --- | --- | --- | --- | --- | --- | --- | --- |
|  | 10.035 | 0.12 | C_9_H_18_ | Alkane | Cyclohexane, (1-methylethyl)- | 915 | Anticancer , Antioxidant, Antibacterial | Shoaib, M et al., 2019 |
|  | 10.750 | 0.30 | C_9_H_18_O | Ketone | 2-Nonanone | 0 | Antifungal  Antibacterial | Abarca, R et al.,2017  Orlanda, J. F., & Nascimento, A. R. 2015 |
|  | 10.985 | 0.15 | C_10_H_14_O | Monoterpene | 2-isopropyl-5-methylphenol | 0 | Antibacterial, antifungal, anti-inflammatory, antioxidant | Nagoor Meeran et al.,2017 |
|  | 11.134 | 0.16 | C_9_H_10_O_2_ | Phenol | 4-Hydroxy-3-methylacetophenone | 1363 | Antibacterial, Anti-inflammatory, | He, J. et al., 2022 |
|  | 12.106 | 0.40 | C_12_H_26_O | Fatty alcohol | 1-Dodecanol | 1457 | Antibacterial | Togashi, N et al., 2007 |
|  | 12.208 | 0.16 | C_11_H_24_ | Alkane | Nonane, 3,7-dimethyl- | 0 | Antitumoral Antimicrobial Antioxidative | Shettima, A. Y et al., 2013 |
|  | 13.006 | 0.99 | C_8_H_12_O | Ketone | 6-Methyl-3,5-heptadiene-2-one | 946 | Antimicrobial and anticancerogenic | Güler et al., 2017 |
|  | 14.107 | 0.40 | C_9_H_16_ | Cyclic alkane | Bicyclo[4.3.0]nonane | 981 | Antimicrobial, antitumor | Rajivgandhi et al., 2020 |
|  | 14.635 | 1.06 | C_16_H_34_O | Alcoholic compound | 1-Hexadecanol | 0 | Antimicrobial | Sarada et al. 2011 |
|  | 15.259 | 0.19 | C_19_H_17_NO_3_ | Benzene nitrile | Benzeneheptanenitrile, .zeta.-hydroxy-.beta.,.delta.-dioxo-.zeta.-phenyl | 0 | Antimicrobial | Ponzilacqua et al., 2018 |
|  | 15.994 | 0.31 | C_13_H_16_O_2_ | Phenyl ketone | Methanone, (1-hydroxycyclohexyl)phenyl- | 1740 | Antioxidant, Antibacterial | Gattouche et al., 2020 |
|  | 16.900 | 0.73 | C_19_H_38_ | Alkene | 1-Nonadecene | 1900 | Antibacterial | Heng et al.,2020 |
|  | 17.644 | 1.05 | C_16_H_22_O_4_ | Ester | 1,2-Benzenedicarboxylic acid, bis(2-methylpropyl) ester | 1908 | Antibacterial | Sivakumar, S. R. et al., 2014 |
|  | 18.127 | 0.11 | C_27_H_40_O_4_ | Phthalic acid di-ester | Phthalic acid, isohexyl tridec-2-yn-1-yl ester | 3083 | Acidifier, Arachidonic acid inhibitor, Increase Decarboxylase activity | Kalaivannan et al., 2021 |
|  | 18.262 | 0.41 | C_18_H_36_O_2_ | Ester | Heptadecanoic acid, methyl ester | 0 | Antibacterial | Davoodbasha, M et al., 2018 |
|  | 18.356 | 0.65 | C_18_H_28_O_3_ | Ester | Methyl-3-(3,5-ditertbutyl-4  -hydroxyphenyl) propionate | 0 | Antioxidant | Singh et al., 2017 |
|  | 18.616 | 1.21 | C_18_H_24_O_6_ | Ester | 1,2-benzenedicarboxylic acid, 2-butoxy-2-oxoethyl butyl ester | 0 | Antibacterial | Kumar, A et al., 2022 |
|  | 18.949 | 0.25 | C_15_H_32_O | Alcohol | 1-Pentadecanol | 0 | Antibacterial | Chatterjee, S et al., 2018 |
|  | 19.908 | 0.72 | C_18_H_32_O_2_ | Fatty acid | 9,12-Octadecadienoic acid | 2183 | Antibacterial, anti-inflammatory, antioxidant | Nuerxiati, R., et al., 2021; Mathur, A. et al., 2011 |
|  | 19.967 | 0.92 | C_17_H_30_O_2_ | Fatty acid | Hexadecanoic acid, methyl ester | 0 | Antibacterial, Antifungal, Antidiabetic, Anti-inflammator, Hypocholesterolemic , Nematicide | Pinto et al. 2017 ;Qureshi et al. 2019) |
|  | 20.073 | 0.22 | C_20_H_40_O | Phytol | 3,7,11,15-Tetramethyl-2-hexadecen-1-ol- | 2045 | Anti-inflammatory, antioxidant, antimicrobial, anticancer, anti-diuretic, Antidiabetic ,Immunostimulatory | Ismail et al. 2020 ; Venkata raman et al. 2012 |
|  | 20.194 | 0.29 | C_21_H_42_O_2_ | Fatty acid | Eicosanoic acid, methyl ester | 0 | Antimicrobial, antioxidant, Anti-inflammatory | Elagbar, Z. A et al., 2016; Stillwel, W., et al., 2016 |
|  | 23.174 | 0.15 | C_13_H_24_O_2_ | Fatty acid | Cyclohexaneacetic acid, .alpha.-methyl-.alpha.-propyl-, methyl ester | 1460 | Antimicrobial | Okey-Nzekwe, C et al., 2019 |
|  | 23.322 | 0.13 | C_17_H_31_F_3_O_2_Si | Ester | cis-5-Dodecenoic acid, dimethyl(3,3,3-trifluoropropyl)silyl ester | 1631 | Antimicrobial, antioxidant | Hussein & Hamad 2020 |
|  | 23.417 | 0.16 | C_19_H_34_O_2_ | Fatty acid | 17-Octadecynoic acid, methyl ester | 2075 | Antimicrobial, antioxidant, Antihypertensive | Bhattacharyya, R et al., 2019; Shawer, E. et al., 2022 |
|  | 24.725 | 0.27 | C_20_H_44_O_3_Si | Silane | Silane diethyldodecyloxy (2-ethoxyethyloxy)- | 2147 | Antimicrobial | Okoro, E.E., et al.,2019 |
|  | 25.125 | 0.21 | C_23_H_44_O_2_ | Fatty acid | 13-docosenoic acid, methyl ester | 0 | Antimicrobial | Munged, I et al., 2022 |
|  | 26.781 | 7.05 | C_12_H_18_O_3_ | Cyclic alkane | Spiro[bicyclo[2.2.1]heptane-2,2'-[1,3]dioxolan]-3-one, 1,7,7-trimethyl- | 0 | Antimicrobial | Mohan, J. 2003 |
|  | 27.051 | 0.43 | C_15_H_24_O_2_ | Ketone | Ethanone, 1-[5-hydroxy-2,2,5-trimethyl-7-(1-methylethenyl)bicyclo[4.1.0]hept-1-yl]- | 0 | Antimicrobial | Darekar, N. R. et al., 2022 |
|  | 27.366 | 0.38 | C_13_H_16_O_3_S | Phenol | 2-[(phenylsulfonyl)methylene]cyclohexanol | 0 | Antimicrobial, Analgesic. Antidepressant | Mukhtar et al., 2018 |
|  | 28.051 | 15.26 | C_12_H_18_O_3_ | Cyclic alkane | Spiro[bicyclo[2.2.1]heptane-2,2'-[1,3]dioxolane]-3-one, 1,7,7-trimethyl- | 1493 | Antimicrobial | Mohan, J. 2003 |
|  | 28.138 | 8.06 | C_12_H_14_O3 | Ester | beta.-Phenoxyethyl methacrylate | 1501 | Antibacterial | Meena & Basha 2017 |
|  | 28.237 | 1.51 | C_14_H_22_O6 | Ester | 2-Propenoic acid, 2-methyl-, 1,2-ethanediylbis(oxy-2,1-ethanediyl) ester | 1834 | Antimicrobial | Alva, P et al., 2021 |
|  | 28.465 | 3.63 | C_28_H_36_O_11_ | Triterpenoid | Bruceantin | 3899 | Anticancerous, Antimalarial | Zhao, L et al., 2014; Houël, E et al., 2013 |
|  | 28.728 | 3.16 | C_28_H_36_O_11_ | Triterpenoid | Bruceantin | 3899 | Anticancerous, Antimalarial | Zhao, L et al., 2014; Houël, E et al., 2013 |
|  | 28.863 | 12.69 | C_11_H_24_FO_2_P | Cyclic alkane | 3,5,5-Trimethylhexyl ethylphosphonofluoridate | 0 | Nerve agent | Gilley et al., 2009 |
|  | 29.356 | 3.09 | C_28_H_36_O_11_ | Triterpenoid | Bruceantin | 3899 | Anticancerous, Antimalarial | Zhao, L et al., 2014; Houël, E et al., 2013 |
|  | 29.630 | 2.20 | C_28_H_36_O_11_ | Triterpenoid | Bruceantin | 3899 | Anticancerous, Antimalarial | Zhao, L et al., 2014; Houël, E et al., 2013 |
|  | 29.776 | 1.20 | C_21_H_32_O_2_ | Steroid | 3-Hydroxypregn-5-en-20-one | 0 | Anticancerous, Antibacterial | Choudhary, M et al., 2011; Figueroa Valverde et al., 2008 |
|  | 31.887 | 11.82 | C_21_H_34_O_3_ | Carboxylic acid | 1H-Naphtho[2,1-b]pyran-7-carboxylic acid, 3-ethenyldodecahydro-3,4a,7,10a-tetramethyl-, methyl ester, | 2244 | Antioxidant, Antimicrobial | Suryavanshi et al., 2021 |
|  | 32.110 | 2.74 | C_21_H_34_O_3_ | Carboxylic acid | 1H-Naphtho[2,1-b]pyran-7-carboxylic acid, 3-ethenyldodecahydro-3,4a,7,10a-tetramethyl-, methyl ester, | 2244 | Antioxidant, Antimicrobial | Suryavanshi et al., 2021 |
|  | 33.582 | 9.07 | C_11_H_24_FO_2_P | Cyclic alkane | 3,5,5-Trimethylhexyl ethylphosphonofluoridate | 0 | Nerve agent | Gilley et al., 2009 |
|  | 34.189 | 3.85 | C_7_H_8_O_2_S | Carboxylic acid | 2-Thiabicyclo[3.1.0]hex-3-ene-3-carboxylic acid, methyl ester | 0 | Antibacterial | Rapando et al., 2020 |
|  | 34.789 | 2.16 | C_29_H_52_O_2_ | Pyran | 4H-Pyran-4-one, 2,3-dihydro-6-methyl-2-(14Z)-14-tricosen-1-yl- | 0 | Antifungal. Antioxidant | Teoh, & Mashitah 2016 |

Table 3: Chemical composition of hexane extract from *Ajuga integrifolia*

| **S. No.** | **Retention Time (min.)** | **Area%** | **Formula** | **Nature of Compound** | **Name of Compound** | **Retention index** | **Biological Activity** | **Reference** |
| --- | --- | --- | --- | --- | --- | --- | --- | --- |
|  | 7.087 | 2.98 | C_12_H_26_ | Alkane | 3,7-Dimethyldecane | 1086 | Antimicrobial | Al-rubaye et al., 2020 |
|  | 7.180 | 1.17 | C_11_H_24_ | Alkane | 5-Ethyl-2 methyloctane | 986 | Antibacterial | Saraswathy & Lavanya 2013 |
|  | 7.708 | 0.43 | C_13_H_28_ | Alkane | 5-Isobutylnonane | 1185 | Antiproliferative, Antimicrobial | Vuko, E et al., 2021 |
|  | 7.817 | 2.29 | C_12_H_26_ | Alkane | 3,7-Dimethyldecane | 1086 | Antimicrobial | Al-rubaye et al., 2020 |
|  | 7.907 | 0.96 | C_12_H_26_ | Alkane | 3,7-Dimethyldecane | 1086 | Antimicrobial | Al-rubaye et al., 2020 |
|  | 8.027 | 0.45 | C_12_H_26_ | Alkane | 2,3,6,7-Tetramethyloctane | 958 | Antimicrobial | Ishola, F et al., 2017 |
|  | 8.734 | 0.88 | C_9_H_20_O_2_S | Silane | Silane, cyclohexyldimethoxymethyl- | 1041 | Aldose reductase inhibitory activity | Ibrahim et al., 2022 |
|  | 10.397 | 0.33 | C_14_H_30_O_3_S | Ester | Sulfurous acid, 2-ethylhexyl isohexyl ester | 1908 | Antioxidant and Antibacterial | Arulkumar, A et al., 2018 |
|  | 10.476 | 3.22 | C_14_H_30_ | Alkane | 4,6-Dimethyldodecane | 1285 | Antimicrobial | Añides, J. A. et al., 2019 |
|  | 10.599 | 0.73 | C_14_H_30_ | Alkane | 4,6-Dimethyldodecane | 1285 | Antimicrobial | Añides, J. A. et al., 2019 |
|  | 10.790 | 0.79 | C_14_H_30_ | Alkane | 4,6-Dimethyldodecane | 1285 | Antimicrobial | Añides, J. A. et al., 2019 |
|  | 11.056 | 0.31 | C_9_H_19_ I | Akane | Nonyl iodide | 1330 |  |  |
|  | 11.136 | 2.34 | C_14_H_30_ | Alkane | 4,6-Dimethyldodecane | 1285 | Antimicrobial | Añides, J. A. et al., 2019 |
|  | 11.270 | 1.16 | C_14_H_30_ | Alkane | 4,6-Dimethyldodecane | 1285 | Antimicrobial | Añides, J. A. et al., 2019 |
|  | 11.393 | 1.10 | C_13_H_28_ | Alkane | 5-Methyl-5-propylnonane | 1229 | Antiproliferative, Antimicrobial | Vuko, E et al., 2021 |
|  | 12.992 | 0.47 | C_14_H_42_O_7_Si_7_ | Silane | Cycloheptasiloxane, tetradecamethyl- | 1447 | Antibacterial, immunomodulatory, antitumor, antifungal, antifouling, | Rasyid & Putra 2023 |
|  | 13.297 | 0.27 | C_12_H_25_I | Alkane | Dodecane, 1-iodo- | 1628 | Antimicrobial | Rao, S et al., 2022 |
|  | 13.351 | 2.52 | C_17_H_36_ | Alkane | Heptadecane | 1711 | Antioxidant, Antibacterial, Anti-inflammatory | Chakraborty, D et al., 2023 |
|  | 13.471 | 0.46 | C_14_H_30_ | Alkane | 4,6-Dimethyldodecane | 1285 | Antimicrobial | Añides, J. A. et al., 2019 |
|  | 13.704 | 0.35 | C_30_H_58_O_4_ | Fatty acid | Decanedioic acid, didecyl ester | 0 | Antiseptic, Antimicrobial | Sahithya& Krishnaveni 2022 |
|  | 13.916 | 2.77 | C_14_H_30_ | Alkane | 4,6-Dimethyldodecane | 1285 | Antimicrobial | Añides, J. A. et al., 2019 |
|  | 14.041 | 0.65 | C_14_H_30_ | Alkane | 4,6-Dimethyldodecane | 1285 | Antimicrobial | Añides, J. A. et al., 2019 |
|  | 14.163 | 0.46 | C_14_H_30_ | Alkane | 4,6-Dimethyldodecane | 1285 | Antimicrobial | Añides, J. A. et al., 2019 |
|  | 14.307 | 0.48 | C_13_H_28_ | Alkane | 5-Methyl-5-propylnonane | 1229 | Antiproliferative, Antimicrobial | Vuko, E et al., 2021 |
|  | 15.871 | 2.11 | C_20_H_42_ | Alkane | Eicosane | 2009 | Wound healing, antifungal compounds, Antidiabetic | Chuah et al. 2018;  Pizon et al.,2018 |
|  | 16.357 | 1.53 | C_20_H_42_ | Alkane | Eicosane | 2009 | Wound healing, Antifungal, Antidiabetic | Chuah et al. 2018; Pizon et al.,2018 |
|  | 16.457 | 0.37 | C_24_H_50_ | Alkane | Tetracosane | 0 | Anticancerous, Antioxidant | Akinwumi et al., 2022; Lomarat et al., 2015 |
|  | 16.563 | 0.46 | C_16_H_34_ | Alkane | 2,6,10-Trimethyltridecane | 0 | Antimicrobial, Antioxidant | Faridha et al., 2016 |
|  | 16.664 | 0.60 | C_12_H_36_O_6_Si_6_ | Alkane | Cyclohexasiloxane, dodecamethyl- | 1240 | Antimicrobial, Antiseptic, and skin conditioning | Rasyid & Putra 2023 |
|  | 17.362 | 0.77 | C_20_H_38_ | Diterpene | Neophytadiene | 0 | Anxiolytic-like and anticonvulsant; anti-inflammatory, analgesic, antipyretic, antioxidant, and antimicrobial. | Gonzalez-Rivera et al., 2023; Al-Rajhi et al., 2022 |
|  | 17.645 | 0.59 | C_16_H_22_O_4_ | Fatty acid | 1,2-Benzenedicarboxylic acid, bis(2-methylpropyl) ester | 1908 | Antimicrobial, Antinematicidal | Sholkamy et al., 2020 |
|  | 18.121 | 0.97 | C_20_H_42_ | Alkane | Eicosane | 2009 | Wound healing, Antifungal, Antidiabetic | Chuah et al. 2018; Pizon et al.,2018 |
|  | 18.550 | 0.96 | C_20_H_42_ | Alkane | Eicosane | 2009 | Wound healing, Antifungal, Antidiabetic | Chuah et al. 2018; Pizon et al.,2018 |
|  | 18.725 | 0.33 | C_16_H_34_ | Alkane | 2,6,10-Trimethyltridecane | 0 | Antimicrobial, Antioxidant | Faridha et al., 2016 |
|  | 18.937 | 0.62 | C_24_H_48_O_2_ | Fatty acid | Docosanoic acid, ethyl ester | 2574 | Antimicrobial, Antioxidant | Chenniappan, J et al., 2020 |
|  | 20.074 | 4.26 | C_20_H_40_O | Diterpene | Phytol | 2045 | Antimicrobial, Antioxidant; Anti-inflammatory, Antiarthritic, Antihyperalgesic | Saha & Bandyopadhyay 2020 ; Carvalho et al., 2020 |
|  | 20.537 | 0.62 | C_20_H_42_ | Alkane | Eicosane | 2009 | Wound healing, Antifungal, Antidiabetic | Chuah et al. 2018; Pizon et al.,2018 |
|  | 21.739 | 0.53 | C_21_H_44_ | Alkane | Heneicosane | 2109 | Antimicrobial, Anti-inflammatory, Antipyretic, Analgesic | Vanitha et al., 2020; Okechukwu, P. N. 2020 |
|  | 22.002 | 0.38 | C_24_H_50_ | Alkane | Tetracosane | 0 | Anticancerous, Antioxidant | Akinwumi et al., 2022; Lomarat et al., 2015 |
|  | 22.349 | 0.52 | C_21_H_44_ | Alkane | 5,5-Diethylheptadecane | 2024 | Anti-inflammatory, Antimicrobial, Antioxidant | Chakraborty et al., 2023 |
|  | 22.577 | 0.55 | C_21_H_44_ | Alkane | Heneicosane | 2109 | Antimicrobial, Anti-inflammatory, Antipyretic, Analgesic | Vanitha et al., 2020; Okechukwu, P. N. 2020 |
|  | 23.317 | 0.25 | C_19_H_17_ClO_4_ | Ester | Succinic acid, 3-chlorophenyl 3-phenylprop-2-en-1-yl ester | 0 | Antimicrobial | Daji et al., 2023 |
|  | 23.380 | 0.71 | C_20_H_42_ | Alkane | Eicosane | 2009 | Wound healing, Antifungal, Antidiabetic | Chuah et al. 2018; Pizon et al.,2018 |
|  | 24.154 | 1.08 | C_21_H_44_ | Alkane | Heneicosane | 2109 | Antimicrobial, Anti-inflammatory, Antipyretic, Analgesic | Vanitha et al., 2020; Okechukwu, P. N. 2020 |
|  | 25.543 | 0.29 | C_24_H_48_O_2_ | Ester | Heneicosanoic acid, 3,3-dimethyl-, methyl ester | 2490 | Antimicrobial | Khromykh et al., 2022 |
|  | 25.620 | 0.86 | C_44_H_90_ | Alkane | Tetratetracontane | 4395 | Antimicrobial, Anticancer | Asnaashari et al., 2019; Ibnouf et al., 2022 |
|  | 25.737 | 0.39 | C_30_H_50_ | Terpenoid | Squalene | 2914 | Antioxidant, moisturizer, detoxifying and anticancer agent | Lou‐Bonafonte et al., 2018; Lozano-Grande et al., 2018 |
|  | 26.325 | 1.11 | C_40_H_82_ | Alkane | Tetracontane | 3997 | Anti-inflammatory, Antibacterial and Analgesic | Roopa et al., 2020 |
|  | 26.777 | 1.77 | C_13_H_16_O_2_ | Ketone | 5-Methyl-1-phenyl-2,4-hexanedione | 1598 |  |  |
|  | 27.068 | 18.72 | C_15_H_26_O | Sesquiterpene | Ledol | 1530 | Antioxidant, an-timicrobial, anti-fungus activity, hepatoprotective activity | Koirala et al., 2020 |
|  | 27.211 | 1.51 | C_16_H_24_O_3_ |  | No name | 0 |  |  |
|  | 27.964 | 2.51 | C_40_H_82_ | Alkane | Tetracontane | 3997 | Anti-inflammatory, Antibacterial and Analgesic | Roopa et al., 2020 |
|  | 28.050 | 7.23 | C_13_H_16_O_2_ | Ketone | 5-Methyl-1-phenyl-2,4-hexanedione | 1598 | Antioxidant, antidiabetic | Suryavanshi et al., 2021 |
|  | 28.385 | 0.75 | C_29_H_50_O_2_ | Ketone | Vitamin E | 3149 | Antioxidant, moisturizer, Anti-inflammatory, Immune booster, Anticancerous | Liao et al., 2022; Shahidi et al., 2021 |
|  | 28.972 | 1.30 | C_25_H_50_Br_2_ | Alkane | Erythro-9,10-Dibromopentacosane | 2970 | Anti-inflammatory, antioxidant, anti-ulcer, cardiotonic, and anticancer | Piskov et al., 2022 |
|  | 29.931 | 3.12 | C_29_H_46_O | Steroid | Pregn-5-en-3-ol, 20-methyl-21- | 0 | Antioxidant, Anti-inflammatory, Antilipidemic, Antidiabetic, and Anticancer | Varghese et al., 2021 |
|  | 30.174 | 2.76 | C_40_H_82_ | Alkane | Tetracontane | 3997 | Anti-inflammatory, Antibacterial and Analgesic | Roopa et al., 2020 |
|  | 30.534 | 1.66 | C_29_H_48_O | Phytosterol | Stigmasterol | 2739 | Antioxidant, Anti-inflammatory, and Anticancer | Ahamed et al., 2022 |
|  | 31.505 | 1.26 | C_42_H_63_O_3_P | Phenol | Phenol, 2,4-bis(1,1-dimethylethyl)-, phosphite | 0 | Anti-enterococcal, anti-microbial, and anti-oxidant | Tyagi et al., 2021 |
|  | 31.639 | 4.22 | C_33_H_54_O_3_ | Steroid | Cholest-22-ene-21-ol, 3,5-dehydro-6-methoxy-, pivalate | 2973 | [Antimicrobial](https://www.sciencedirect.com/topics/neuroscience/antimicrobials), anti-inflammatory, antiarthritic, antidiuretic and antiasthmatic | Albratty et al., 2021 |
|  | 31.866 | 2.01 | C_23_H_34_O_4_ | Steroid | 21-Acetoxypregnenolone | 2630 | Anticancer | Majolo et al., 2020 |
|  | 32.090 | 0.56 | C_21_H_34_O_3_ | Carboxylic acid | 1H-Naphtho[2,1-b]pyran-7-carboxylic acid, 3-ethenyldodecahydro-3,4a,7,10a-tetramethyl-, methyl ester, | 0 | Antioxidant, Antimicrobial | Suryavanshi et al., 2021 |
|  | 33.328 | 2.08 | C_40_H_82_ | Alkane | Tetracontane | 3997 | Anti-inflammatory, Antibacterial and Analgesic | Roopa et al., 2020 |

Table 4: Chemical composition of water extract from *Ajuga integrifolia*

| **S. No.** | **Retention Time (min.)** | **Area%** | **Formula** | **Nature of Compound** | **Name of Compound** | **Retention index** | **Biological Activity** | **Reference** |
| --- | --- | --- | --- | --- | --- | --- | --- | --- |
|  | 4.721 | 3.98 | C_2_H_8_N_2_ | Amine | Hydrazine, 1,1-dimethyl | 0 | Antifungal, Antibacterial | Jaibangyang et al., 2021 |
|  | 4.863 | 1.04 | C_4_H_6_O_2_ | Furan | 2(3H)-Furanone, dihydro- | 0 | Antimicrobial | Abou-Elmagd et al.,2015 |
|  | 4.993 | 0.96 | C_7_H_16_O | Alcohol | 3,3-Dimethyl-2-pentanol | 795 | Anticancerous | Hayton, C et al., 2023 |
|  | 5.085 | 3.30 | C_5_H_6_O_2_ | Ketone | 2-Cyclopenten-1-one, 2-hydroxy- | 883 | Antioxidant, Antimicrobial | Wang, J et al., 2015 |
|  | 5.954 | 1.45 | C_6_H_8_O_4_ | Ketone | 2,4-Dihydroxy-2,5-dimethyl-3(2H)-furan-3-one | 1173 | Antioxidant, Gustatory activity, flavoring agents | Sakika et al., 2022; Chukwu et al., 2017 |
|  | 6.222 | 1.05 | C_4_H_6_O_3_ | Lactone | 2-Hydroxy-gamma-butyrolactone | 1013 | Antifungal, Antibacterial, Insecticidal | Bharali et al., 2017 |
|  | 6.503 | 3.25 | C_3_H_8_O_3_ | Alcohol | Glycerin | 967 | Antimicrobial | Yassin et al., 2021 |
|  | 7.032 | 0.76 | C_5_H_6_O_3_ | Ketone | 4,5-Dimethyl-1,3-dioxol-2-one | 975 | Antihypertensive | Abu Bakar et al., 2015 |
|  | 7.284 | 1.51 | C_6_H_8_O_3_ | Furan | 2,5-Anhydro-1,6-dideoxyhexo-3,4-diulose | 0 | Antioxidant | Krupa et al., 2022 |
|  | 7.852 | 1.27 | C_5_H_10_O | Aldehyde | Pentanal | 0 | Antifungal | Li, B et al., 2022 |
|  | 8.523 | 1.42 | C_6_H_8_O_4_ | Lactone | 2-acetyl-2-hydroxy-.gamma.butyrolactone | 0 | Antifungal, Antibacterial, Insecticidal | Bharali et al., 2017 |
|  | 8.669 | 10.31 | C_6_H_8_O_4_ | Ketone | 4H-Pyran-4-one, 2,3-dihydro-3,5-dihydroxy-6-methyl- | 1269 | Antimicrobial, Antiinflammatory | Syed et al., 2014 |
|  | 8.908 | 0.59 | C_12_H_18_O_6_ | Fatty acid | Decanedioic acid, 3,8-dioxo-, dimethyl ester | 0 | Antimicrobial | Jaradat et al., 2021 |
|  | 9.210 | 0.65 | C_8_H_18_O_3_ | Ether | Ethanol, 2-(2-butoxyethoxy)- | 0 | Larvicidal, stabilizer in pesticides & fertilizers | Chan et al., 2022 |
|  | 9.379 | 0.32 | C_14_H_30_ | Alkane | Tridecane | 1313 | Antioxidant, Antimicrobial | More et al., 2022 |
|  | 9.541 | 0.26 | C_7_H_12_O_5_ | Alcohol | Glycerol 1,2-diacetate | 1230 | Antifungal, Antibacterial | Balachandar et al., 2022 |
|  | 9.726 | 0.30 | C_6_H_8_O_4_ | Carbohydrate | 1,4:3,6-Dianhydro-.alpha.-d-glucopyranose | 916 | Acute neurologic disorders treatment, Phobic disorders treatment | Ragupathi et al., 2018 |
|  | 9.880 | 1.43 | C_8_H_8_O | Benzofuran | 2,3-Dihydro-benzofuran | 0 | Antioxidant, Antitumor and Antiproliferative | Nousheen et al., 2022 |
|  | 10.214 | 1.27 | C_5_H_10_O_4_ | Alcohol | 1,2,3-Propanetriol, 1-acetate | 1091 | Antibacterial | Saeed et al., 2023 |
|  | 10.803 | 3.65 | C_6_H_6_O_2_ | Phenol | Hydroquinone | 1122 | Antibacterial, Anticancerous | Byeon et al., 2018 |
|  | 11.114 | 2.68 | C_9_H_10_O_2_ | Phenol | 2-methoxy-4-vinylphenol | 0 | Antimicrobial, antioxidant, anti-inflammatory, analgesic | Rubab et al.,2020 |
|  | 11.480 | 1.96 | C_9_H_9_NO | Phenol | 3,5-Dimethylphenyl isocyanate | 0 | Antibacterial | Nafis et al., 2021 |
|  | 11.609 | 1.53 | C_9_H_11_NO | Pyridine | 2-(2,3-Dimethyl-2-oxiranyl) pyridine | 0 | Antibacterial | Mudhafar et al., 2023 |
|  | 11.727 | 0.68 | C_7_H_8_O_2_ | Alcohol | 1,2-Benzenediol, 3-methyl | 1235 | Antimicrobial | Kim & Lee 2014 |
|  | 12.211 | 0.23 | C_15_H_32_ | Alkane | Pentadecane | 0 | Anti-inflammatory, analgesic, antibacterial and antipyretic | Okechukwu 2020 |
|  | 12.313 | 1.65 | C_9_H_12_O_3_ | Carboxylic Acid | Cyclohexanecarboxylic acid, 3-methylene-2-oxo-methyl ester | 0 | Antimicrobial, anti-inflammatory, | Mujeeb et al., 2014 |
|  | 12.968 | 0.36 | C_9_H_9_NO | Phenol | 2,3-Dimethylphenyl isocyanate | 0 | Antibacterial | Nafis et al., 2021 |
|  | 13.217 | 0.77 | C_10_H_13_NO | Benzofuran | 5-Amino-2,2-dimethyl-2,3-dihydrobenzofuran | 1482 | Antioxidant, Antitumor and Antiproliferative | Nousheen et al., 2022 |
|  | 13.531 | 2.19 | C_10_H_12_O_2_ | Alkene | 4-Cyclopentene-1,3-dione, 4-(3-methyl-2-butenyl)- | 1397 | Antifungal, Antibacterial | Mokhtari et al., 2018 |
|  | 14.230 | 2.35 | C_10_H_11_N_3_O_2_ | Carboxylic acid amide | Acetamide, N-(4-oxo-1,4-dihydro-2H-quinazolin-3-yl)- | 2132 | Antbacterial, Antioxidant | Pandiyan et al., 2019 |
|  | 14.488 | 2.63 | C_10_H_11_N_3_O_2_ | Carboxylic acid amide | Acetamide, N-(4-oxo-1,4-dihydro-2H-quinazolin-3-yl)- | 2132 | Antbacterial, Antioxidant | Pandiyan et al., 2019 |
|  | 14.861 | 3.89 | C_8_H_8_O_4_ | Carboxylic acid | Benzeneacetic acid, 2,5-dihydroxy- | 0 | Antibacterial | Rohatgi & Gupta (2023). |
|  | 15.355 | 6.92 | C_7_H_12_O_6_ | Carboxylic acid | 1,3,4,5-Tetrahydroxy-cyclohexanecarboxylic acid | 0 | Antbacterial, Antioxidant | Chakraborty et al., 2021 |
|  | 15.829 | 0.31 | C_13_H_20_O_2_ | Alcohol | 4,6,10,10-Tetramethyl-5-oxatricyclo[4.4.0.0(1,4)]dec-2-en-7-ol | 1457 | Decrease endothelial leucocyte and platelet adhesion, fertility enhancer | Rao & Lakshmi (2018) |
|  | 16.869 | 1.13 | C_11_H_16_O_3_ | Benzofuran | 2(4H)-Benzofuranone, 5,6,7,7a-tetrahydro-6-hydroxy-4,4,7a-trimethyl | 0 | Antbacterial, Antioxidant | Sutarman et al.,2021 |
|  | 17.639 | 0.56 | C_8_H_10_N_4_O_2_ | Methyl xanthines | Caffeine | 1795 | Antidepressant, antioxidant, stimulant and anti-inflammatory | Hall et al., 2015 |
|  | 18.265 | 0.73 | C_17_H_34_O_2_ | Fatty acid | Hexadecanoic acid, methyl ester | 1878 | Antbacterial, Antifungal | Abubakar & Majinda (2016) |
|  | 18.359 | 1.58 | C_18_H_28_O_3_ | Carboxylic acid | Benzenepropanoic acid, 3,5-bis(1,1-dimethylethyl)-4-hydroxy-, methyl ester | 2134 | Antifungal, Antibacterial, Antioxidant | Kumar et al., 2016 |
|  | 18.530 | 0.71 | C_9_H_12_N_2_O_2_ | Phenol | l-Tyrophanamide | 1801 | Antioxidant, antidiabetic | Tunna et al., 2015 |
|  | 18.657 | 0.70 | C_9_H_9_NOS_2_ | Thiazole | Benzothiazole, 2-(2-hydroxyethylthio) | 1890 | Antioxidant, anticancerous | Mohammad et al., 2023 |
|  | 19.906 | 0.47 | C_19_H_34_O_2_ | Fatty acid | 9,12-Octadecadienoic acid, methyl ester | 2093 | Antibacterial, anti-inflammatory, antioxidant | Nuerxiati, R., et al., 2021; Mathur, A. et al., 2011 |
|  | 19.966 | 0.51 | C_19_H_36_O_2_ | Fatty acid | 9-Octadecenoic acid (Z)-, methyl ester | 2085 | Antibacterial, anti-inflammatory, antioxidant | Nuerxiati, R., et al., 2021; Mathur, A. et al., 2011 |
|  | 20.198 | 0.25 | C_21_H_42_O_2_ | Fatty acid | Eicosanoic acid, methyl ester | 0 | Antimicrobial, Antioxidant | Huang et al., 2010 |
|  | 20.657 | 0.15 | C_11_H_13_NO_4_ | Phenol | N-methyl-3,4-methylenedioxyphenylalanine | 1927 | Antimicrobial | Morel et al., 2005 |
|  | 22.486 | 0.40 | C_22_H_42_O_4_ | Carboxylic acid | Hexanedioic acid, bis(2-ethylhexyl) ester | 0 | Antbacterial, Antioxidant, anticancer | Hussein et al., 2020 |
|  | 23.531 | 0.40 | C_19_H_38_O_4_ | Fatty acid | Hexadecanoic acid, 2-hydroxy-1-(hydroxymethyl)ethyl ester | 2498 | Antbacterial, Antifungal | Abubakar & Majinda (2016) |
|  | 24.953 | 0.52 | C_21_H_40_O_4_ | Fatty acid | 9-Octadecenoic acid (Z)-, 2-hydroxy-1-(hydroxymethyl)ethyl ester | 2705 | Antibacterial, anti-oxidant, anti-inflammatory and hypocholesterolemic | Christiana et al., 2019 |
|  | 25.110 | 0.41 | C_21_H_42_O_4_ | Fatty acid | Octadecanoic acid, 2,3-dihydroxypropyl ester | 2681 | Anticancer, antimicrobial | Arora et al., 2017 |
|  | 26.177 | 1.78 | C_12_H_14_O_3_ | Phenol | beta.-Phenoxyethyl methacrylate | 1501 | Antibacterial | Hu, X et al., 2023 |
|  | 26.781 | 0.85 | C_12_H_18_O_3_ | Cyclic alkane | Spiro[bicyclo[2.2.1]heptane-2,2'-[1,3]dioxolan]-3-one, 1,7,7-trimethyl- | 0 | Antimicrobial | Mohan, J. 2003 |
|  | 27.683 | 0.27 | C_12_H_24_FO_2_P | Cyclic alkane | 2-tert-Butylcyclohexyl ethylphosphonofluoridate | 0 | Nerve agent | Gilley et al., 2009 |
|  | 28.055 | 8.62 | C_13_H_16_O_2_ | Ketone | 2,4-Hexanedione, 5-methyl-1-phenyl- | 1598 | Antimicrobial | Feng et al., 2021 |
|  | 28.139 | 3.10 | C_13_H_16_O_2_ | Ketone | 2,4-Hexanedione, 5-methyl-1-pheny | 1598 | Antimicrobial | Feng et al., 2021 |
|  | 28.865 | 5.65 | C_12_H_18_O_3_ | Cyclic alkane | Spiro[bicyclo[2.2.1]heptane-2,2'-[1,3]dioxolan]-3-one, 1,7,7-trimethyl- | 1493 | Antimicrobial | Mohan, J. 2003 |
|  | 29.188 | 0.33 | C_20_H_32_O_2_ | Alkane | 2.Beta.-hydroxy-9-oxoverrucosane | 0 | Antibacterial | Sareer et al., 2014 |
|  | 31.874 | 2.71 | C_21_H_34_O_3_ | Carboxylic acid | 1H-Naphtho[2,1-b]pyran-7-carboxylic acid, 3-ethenyldodecahydro-3,4a,7,10a-tetramethyl-, methyl ester | 2244 | Antioxidant, Antimicrobial, Antidiabetic | Suryavanshi et al., 2021 |
|  | 32.100 | 0.68 | C_21_H_34_O_3_ | Carboxylic acid | 1H-Naphtho[2,1-b]pyran-7-carboxylic acid, 3-ethenyldodecahydro-3,4a,7,10a-tetramethyl-, methyl ester | 2244 | Antioxidant, Antimicrobial, Antidiabetic | Suryavanshi et al., 2021 |
|  | 33.577 | 0.660 | C_14_H_24_O_3_ | Ketone | 3,6,6-Trimethylundecane-2,5,10-trione | 1672 | Antioxidant, antidiabetic | Suryavanshi et al., 2021 |

Table 5: LCMS of methanol extract from *Ajuga integrifolia*

| **S.NO.** | **Name** | **Formula** | **RT (Min)** | **Area(max.)** | **Cal MW.** | **m/z** |
| --- | --- | --- | --- | --- | --- | --- |
|  | Corchorifatty acid F | C18 H32 O5 | 12.75 | 1205790900 | 328.22392 | 327.21664 |
|  | Corchorifatty acid F | C18 H32 O5 | 12.01 | 1116832689 | 328.22405 | 327.21677 |
|  | (15Z)-9,12,13-Trihydroxy-15-octadecenoic acid | C18 H34 O5 | 13.57 | 953025930.3 | 330.23949 | 329.23224 |
|  | Glycitein | C16 H12 O5 | 15.464 | 870961726.4 | 284.06754 | 283.06027 |
|  | (+/-)9-HpODE | C18 H32 O4 | 16.813 | 793875948.6 | 312.22912 | 311.22187 |
|  | MFCD00041919 | C19 H32 O3 S | 23.964 | 737195143 | 340.20598 | 339.1987 |
|  | CHEMBRDG-BB 9071407 | C9 H11 F O S | 0.684 | 542619976.5 | 186.05144 | 167.03354 |
|  | 4,5-Dicaffeoylquinic acid | C25 H24 O12 | 9.42 | 533229174.2 | 516.12519 | 515.11792 |
|  | 2-(3,4-Dihydroxyphenyl)ethyl 3-O-(6-deoxy-β-L-mannopyranosyl)-6-O-[(2E)-3-(3,4-dihydroxyphenyl)-2-propenoyl]-β-D-glucopyranoside | C29 H36 O15 | 8.312 | 436180437.2 | 624.20355 | 623.19629 |
|  | 2-Methyl-2-propanyl 4-[(3R)-3-{[(benzyloxy)carbonyl]amino}-4-methoxy-4-oxobutyl]-1-piperidinecarboxylate | C23 H34 N2 O6 | 22.609 | 351900746.3 | 434.24183 | 433.23456 |
|  | L6X660925G | C23 H32 N2 O6 | 20.965 | 340654338.9 | 432.22606 | 431.21878 |
|  | (+/-)9-HpODE | C18 H32 O4 | 16.041 | 329783773.6 | 312.22923 | 311.22198 |
|  | (+/-)9-HpODE | C18 H32 O4 | 16.371 | 299770457.7 | 312.2292 | 311.22195 |
|  | Glycitein | C16 H12 O5 | 14.984 | 296650738.8 | 284.06761 | 283.06033 |
|  | 2-(3,4-Dihydroxyphenyl)ethyl 3-O-(6-deoxy-β-L-mannopyranosyl)-6-O-[(2E)-3-(3,4-dihydroxyphenyl)-2-propenoyl]-β-D-glucopyranoside | C29 H36 O15 | 8.765 | 296429405.1 | 624.20333 | 623.19604 |
|  | 4,5-Dicaffeoylquinic acid | C25 H24 O12 | 8.838 | 276191872.9 | 516.12514 | 515.11786 |
|  | Glycitein | C16 H12 O5 | 14.614 | 267848408.9 | 284.06755 | 283.06027 |
|  | 16-Hydroxyhexadecanoic acid | C16 H32 O3 | 25.035 | 260834720.5 | 272.23436 | 271.22708 |
|  | 4,5-Dicaffeoylquinic acid | C25 H24 O12 | 8.701 | 244925231.7 | 516.1252 | 515.11792 |
|  | MFCD00041919 | C19 H32 O3 S | 24.245 | 241475814.1 | 340.20616 | 339.19888 |
|  | (10E,15Z)-9,12,13-Trihydroxy-10,15-octadecadienoic acid | C18 H32 O5 | 14.325 | 237163611.3 | 328.22395 | 327.21671 |
|  | 6,6'-(1,4,10,13-Tetraoxa-7,16-diazacyclooctadecane-7,16-diyl)bis(7H-purine) | C22 H30 N10 O4 | 12.078 | 236414002.7 | 498.24525 | 497.23798 |
|  | L-Seryl-L-lysyl-L-valyl-L-prolyl-L-proline | C24 H42 N6 O7 | 8.179 | 230698003.4 | 526.31276 | 525.30548 |
|  | Corchorifatty acid F | C18 H32 O5 | 13.795 | 229903918 | 328.22403 | 327.21677 |
|  | Ethyl N~2~-(3,4-dimethoxybenzyl)-N~5~-{[(2-methyl-2-propanyl)oxy]carbonyl}-L-ornithinate | C21 H34 N2 O6 | 24.038 | 228624977.5 | 410.24193 | 409.23465 |
|  | Corchorifatty acid F | C18 H32 O5 | 12.928 | 226147785 | 328.22404 | 327.2168 |
|  | (15Z)-9,12,13-Trihydroxy-15-octadecenoic acid | C18 H34 O5 | 15.388 | 218128915.8 | 330.23951 | 329.23228 |
|  | 5-Amino-1,2-oxazole-4-carbonitrile | C4 H3 N3 O | 0.686 | 198021389.3 | 109.02734 | 108.02007 |
|  | 5-Amino-1,2-oxazole-4-carbonitrile | C4 H3 N3 O | 1.386 | 187382549.4 | 109.02733 | 108.02006 |
|  | Corchorifatty acid F | C18 H32 O5 | 14.063 | 180389053.7 | 328.22408 | 327.21681 |
|  | (15Z)-9,12,13-Trihydroxy-15-octadecenoic acid | C18 H34 O5 | 14.538 | 177393873.6 | 330.23965 | 329.23239 |
|  | S-Methyl 4-fluorobutanethioate | C5 H9 F O S | 0.694 | 170320835.5 | 136.03584 | 195.04976 |
|  | Cyclo(D-alanyl-D-prolyl-D-alanyl-L-leucyl-D-alpha-aspartyl-L-valyl) | C26 H42 N6 O8 | 9.107 | 166449344.2 | 566.30745 | 565.30017 |
|  | 2-(3,4-Dihydroxyphenyl)ethyl 3-O-(6-deoxy-β-L-mannopyranosyl)-6-O-[(2E)-3-(3,4-dihydroxyphenyl)-2-propenoyl]-β-D-glucopyranoside | C29 H36 O15 | 0.671 | 162513903.3 | 624.20338 | 623.19611 |
|  | 2-Oxo-5-guanidinovalerate | C6 H10 N3 O3 | 1.493 | 160638719.5 | 172.07217 | 171.0649 |
|  | [Similar to: (3beta,5xi,9xi,22beta)-22,24-Dihydroxyolean-12-en-3-yl 6-deoxy-alpha-L-mannopyranosyl-(1-2)-beta-D-galactopyranuronosyl-(1-2)-beta-D-glucopyranosiduronic acid; ΔMass: -320.3670 Da] | C26 H31 N4 O9 P3 | 9.843 | 159641188.3 | 636.13111 | 635.12384 |
|  | (15Z)-9,12,13-Trihydroxy-15-octadecenoic acid | C18 H34 O5 | 13.731 | 155836358.9 | 330.23964 | 329.23236 |
|  | [Similar to: NP-006532; ΔMass: 3.9933 Da] | C21 H32 N6 O7 | 14.616 | 147165400.3 | 480.23436 | 479.22708 |
|  | Hispidulin | C16 H12 O6 | 12.57 | 145558418.7 | 300.06239 | 299.05511 |
|  | (-)-pinellic acid | C18 H34 O5 | 14.292 | 143616297.6 | 330.23947 | 329.23224 |
|  | Corchorifatty acid F | C18 H32 O5 | 13.424 | 141437391.1 | 328.22396 | 327.21671 |
|  | 2,2,14,14-Tetramethyl-8-oxopentadecanedioic acid | C19 H34 O5 | 14.671 | 138585941 | 342.23967 | 341.23239 |
|  | Chlorogenic acid | C16 H18 O9 | 5.122 | 132220446.5 | 354.09413 | 353.08685 |
|  | Corchorifatty acid F | C18 H32 O5 | 13.29 | 127421914.8 | 328.22407 | 327.2168 |
|  | HP8757000 | C14 H10 O5 | 11.572 | 124442207.3 | 258.05191 | 293.02142 |
|  | (15Z)-9,12,13-Trihydroxy-15-octadecenoic acid | C18 H34 O5 | 14.921 | 121383288.5 | 330.23963 | 329.23237 |
|  | Chlorogenic acid | C16 H18 O9 | 0.68 | 118815613.6 | 354.09401 | 353.08673 |
|  | 2-Methyl-2-propanyl 2-{[(1R,2R)-2-{[(2S)-5-carbamimidamido-1-oxo-2-pentanyl]carbamoyl}cyclohexyl]carbonyl}-2-(2-ethoxy-2-oxoethyl)hydrazinecarboxylate | C23 H40 N6 O7 | 9.916 | 112669791.1 | 512.29688 | 557.29504 |
|  | 4H-Chromeno[3,4-c][1,2,5]oxadiazol-4-one 3-oxide | C9 H4 N2 O4 | 0.719 | 111513444 | 204.01768 | 203.01041 |
|  | terbufibrol | C20 H24 O5 | 14.496 | 106888077.9 | 344.16148 | 343.15421 |
|  | 3-Pyridine(~14~C)carboxamide | C5 [14]C H6 N2 O | 1.387 | 105270788.7 | 124.05082 | 123.04354 |
|  | PEGUNIGALSIDASE ALFA | C24 H44 N6 O9 | 20.134 | 101888663.8 | 560.31782 | 559.31055 |
|  | [Similar to: NP-006532; ΔMass: 3.9932 Da] | C21 H32 N6 O7 | 15.402 | 100771764.6 | 480.23427 | 479.22699 |
|  | NP-003433 | C20 H22 O6 | 14.767 | 100722893.4 | 358.14061 | 357.13333 |
|  | 3-Pyridine(~14~C)carboxamide | C5 [14]C H6 N2 O | 0.682 | 98710181.91 | 124.05084 | 123.04356 |
|  | Sinapinic acid | C11 H12 O5 | 7.687 | 98680498.99 | 224.06744 | 223.06017 |
|  | terbufibrol | C20 H24 O5 | 14.786 | 97283143.36 | 344.1613 | 343.15402 |
|  | [Similar to: (3beta,5xi,9xi,22beta)-22,24-Dihydroxyolean-12-en-3-yl 6-deoxy-alpha-L-mannopyranosyl-(1-2)-beta-D-galactopyranuronosyl-(1-2)-beta-D-glucopyranosiduronic acid; ΔMass: -320.3672 Da] | C24 H24 N6 O15 | 9.195 | 94235429.91 | 636.13087 | 635.1236 |
|  | dCt | C18 H28 O4 | 15.112 | 90741558.97 | 308.19784 | 307.19058 |
|  | 4-[(1-Methyl-1H-pyrrol-2-yl)carbonyl]-N-{4-[4-(4-morpholinylcarbonyl)-1-piperidinyl]phenyl}-1-piperazinecarboxamide | C27 H36 N6 O4 | 21.766 | 90214683.21 | 508.27843 | 507.27115 |
|  | [Similar to: Glycitein; ΔMass: 176.0309 Da] | C18 H16 N6 O9 | 11.692 | 89228660.17 | 460.09938 | 459.0921 |
|  | [Similar to: Glycitein; ΔMass: 176.0311 Da] | C18 H16 N6 O9 | 11.095 | 88974483.58 | 460.09959 | 459.09232 |
|  | Dibutyl Fumarate | C12 H20 O4 | 13.278 | 88839675.65 | 228.1351 | 227.12782 |
|  | T-2 triol | C20 H30 O7 | 9.974 | 87704393.95 | 382.19804 | 381.19077 |
|  | (2'R,3R,4'R,4a'R,5S,8a'S)-5-(3-Furyl)-4'-hydroxy-4a',5'-bis(hydroxymethyl)-2'-methyl-3',4,4',4a',5,7',8',8a'-octahydro-2'H-spiro[furan-3,1'-naphthalen]-2-one | C20 H26 O6 | 13.17 | 85662606.37 | 362.1721 | 361.16483 |
|  | L-Alanyl-L-valylglycyl-L-isoleucylglycyl-L-alanine | C21 H38 N6 O7 | 21.121 | 84776392.14 | 486.28129 | 485.27402 |
|  | Corchorifatty acid F | C18 H32 O5 | 14.444 | 83053551.18 | 328.22398 | 327.21671 |
|  | L-Tyrosylglycylglycyl-L-tryptophyl-L-leucine | C30 H38 N6 O7 | 19.036 | 81334494.04 | 594.27864 | 593.27136 |
|  | (15Z)-9,12,13-Trihydroxy-15-octadecenoic acid | C18 H34 O5 | 14.725 | 80469446.67 | 330.23966 | 329.23242 |
|  | 3,3',3'',3'''-(1,2-Ethanediyldinitrilo)tetrapropanamide | C14 H28 N6 O4 | 10.802 | 78769686.99 | 344.21882 | 343.21155 |
|  | 5'-O-[(2E,6S,7R)-6,7-Dihydroxy-3,7,11-trimethyl-2,10-dodecadien-1-yl]-5,6-dihydrouridine | C24 H40 N2 O8 | 23.07 | 76974776.22 | 484.27858 | 483.2713 |
|  | 13(S)-HOTrE | C18 H30 O3 | 19.109 | 71009579.53 | 294.21871 | 293.21143 |
|  | Thianaphthene-2-boronic acid | C8 H7 B O2 S | 5.141 | 70547650.91 | 178.02525 | 177.01797 |
|  | 2-Fluoro-4-(methylsulfanyl)-1-propoxybenzene | C10 H13 F O S | 1.974 | 66539111.39 | 200.06724 | 199.05997 |
|  | Tetrahydro-4-(4,4,5,5-tetramethyl-1,3,2-dioxaborolan-2-yl)-2H-thiopyran | C11 H21 B O2 S | 13.587 | 65736519.49 | 228.13498 | 227.1277 |
|  | 2-(3-CARBOXYPROPIONYL)-6-HYDROXY-CYCLOHEXA-2,4-DIENE CARBOXYLIC ACID | C11 H12 O6 | 5.253 | 60906454.63 | 240.06244 | 239.05516 |
|  | (2'R,3R,4'R,4a'R,5S,8a'S)-5-(3-Furyl)-4'-hydroxy-4a',5'-bis(hydroxymethyl)-2'-methyl-3',4,4',4a',5,7',8',8a'-octahydro-2'H-spiro[furan-3,1'-naphthalen]-2-one | C20 H26 O6 | 13.657 | 59427636.42 | 362.17192 | 361.16464 |
|  | (15Z)-9,12,13-Trihydroxy-15-octadecenoic acid | C18 H34 O5 | 15.14 | 58014177.53 | 330.23962 | 329.23239 |
|  | Crocetin | C20 H24 O4 | 18.331 | 57892513.79 | 328.16649 | 327.15921 |
|  | Macelignan | C20 H24 O4 | 20.958 | 57512537.05 | 328.1663 | 327.15903 |
|  | CHEMBRDG-BB 9071407 | C9 H11 F O S | 0.963 | 56090381.5 | 186.05161 | 185.04422 |
|  | 2-Fluoro-4-(methylsulfanyl)-1-propoxybenzene | C10 H13 F O S | 1.498 | 55892562.38 | 200.06724 | 199.05997 |
|  | Diethyl (4E)-2-chloro-2,7-dipropyl-4-octenedioate | C18 H31 Cl O4 | 16.521 | 53878028.12 | 346.19008 | 345.1828 |
|  | (2R,3R,4S,5R)-2-(6-Amino-2-{[(2S)-1-(4-methoxyphenyl)-2-propanyl]amino}-9H-purin-9-yl)-5-(2-ethyl-2H-tetrazol-5-yl)tetrahydro-3,4-furandiol | C22 H28 N10 O4 | 11.725 | 53064969.22 | 496.22948 | 495.2222 |
|  | 4,6-Diazido-1,3,5-triazin-2-amine | C3 H4 N10 | 0.674 | 51724779.68 | 180.06196 | 179.05469 |
|  | pyrazinamide | C5 H5 N3 O | 0.683 | 51063321.32 | 123.04302 | 122.03574 |
|  | 2-Oxo-5-guanidinovalerate | C6 H10 N3 O3 | 0.69 | 50783958.27 | 172.0721 | 171.0648 |
|  | 13(S)-HpOTrE | C18 H30 O4 | 17.438 | 49055585.8 | 310.21342 | 345.18277 |
|  | Diethyl 2,2'-{oxybis[2,1-ethanediylcarbamoyl(2,4-dioxo-3,4-dihydropyrimidine-5,1(2H)-diyl)]}diacetate | C22 H28 N6 O11 | 10.363 | 48851609.94 | 552.18276 | 551.17548 |
|  | 4-({5-[(2-aminoethyl)amino]-2,4-dinitrophenyl}amino)-TEMPO | C17 H28 N6 O5 | 12.752 | 48389690.32 | 396.21092 | 395.20364 |
|  | [Similar to: NP-014604; ΔMass: 30.0093 Da] | C17 H28 N10 O5 | 9.633 | 48052723.44 | 452.2245 | 497.22278 |
|  | 4-({5-[(2-aminoethyl)amino]-2,4-dinitrophenyl}amino)-TEMPO | C17 H28 N6 O5 | 11.551 | 48011169.24 | 396.21272 | 395.20544 |
|  | Ethyl (6-amino-4-{[5-(diethylamino)-2-pentanyl]amino}-5-nitro-2-pyridinyl)carbamate | C17 H30 N6 O4 | 11.795 | 47462223.8 | 382.23444 | 381.22723 |
|  | 2-Methyl-2-propanyl 4-[(3R)-3-{[(benzyloxy)carbonyl]amino}-4-methoxy-4-oxobutyl]-1-piperidinecarboxylate | C23 H34 N2 O6 | 22.188 | 46912993.71 | 434.24196 | 433.23468 |
|  | [Similar to: 10-Nitrolinoleate; ΔMass: 181.0377 Da] | C20 H39 N6 O7 P | 20.227 | 46816649.75 | 506.26304 | 505.25577 |
|  | Ethyl 1-{3-[5-(4-morpholinylmethyl)-1H-tetrazol-1-yl]propanoyl}-2-piperidinecarboxylate | C17 H28 N6 O4 | 16.816 | 46715231.01 | 380.2162 | 379.20892 |
|  | Decylubiquinone | C19 H30 O4 | 18.235 | 46433255.09 | 322.21342 | 321.20615 |
|  | N2,N4,N6-Tripropionyloxy-methyl-N2,N4,N6-trimethylmelamine | C18 H30 N6 O6 | 12.307 | 44506253.48 | 426.22417 | 425.21689 |
|  | (2'R,3R,4'R,4a'R,5S,8a'S)-5-(3-Furyl)-4'-hydroxy-4a',5'-bis(hydroxymethyl)-2'-methyl-3',4,4',4a',5,7',8',8a'-octahydro-2'H-spiro[furan-3,1'-naphthalen]-2-one | C20 H26 O6 | 14.336 | 44343892.36 | 362.17195 | 361.16467 |
|  | pyrazinamide | C5 H5 N3 O | 1.792 | 44206257.38 | 123.04303 | 122.03575 |
|  | 5-[6-(Dimethylamino)-9H-purin-9-yl]pentyl 6-[6-(dimethylamino)-9H-purin-9-yl]hexanoate | C25 H36 N10 O2 | 9.478 | 43745891.26 | 508.3022 | 507.29492 |
|  | Oleoyl-L-α-lysophosphatidic acid | C21 H41 O7 P | 24.797 | 43701978.93 | 436.25761 | 435.25034 |
|  | NP-005870 | C20 H26 O5 | 11.926 | 42812060.37 | 346.17695 | 345.16968 |
|  | L6X660925G | C23 H32 N2 O6 | 20.605 | 42404625.87 | 432.22615 | 431.21887 |
|  | L-Seryl-L-threonyl-L-glutaminyl-L-prolyl-L-leucine | C23 H40 N6 O9 | 9.789 | 41807977.04 | 544.28686 | 589.2851 |
|  | 1,1'-(1,3-dimethyl-2-oxoimidazolidine-4,5-diyl)bis(3-tert-butylurea) | C15 H30 N6 O3 | 16.146 | 41016725.36 | 342.23948 | 341.23221 |
|  | 1-(4-Carbamimidamidobutanoyl)-D-prolyl-N-propyl-L-alpha-asparagine | C17 H30 N6 O5 | 13.57 | 40996751.03 | 398.22648 | 397.21921 |
|  | 2-Fluoro-4-(methylsulfanyl)-1-propoxybenzene | C10 H13 F O S | 0.689 | 40570141.36 | 200.06724 | 199.05989 |
|  | N-[5-({3-[(4-Aminobutyl)amino]propyl}carbamoyl)-1-methyl-1H-pyrrol-3-yl]-4-[(N-carbamimidoylglycyl)amino]-1-methyl-1H-pyrrole-2-carboxamide | C22 H36 N10 O3 | 22.334 | 40366029.87 | 488.29701 | 487.28973 |
|  | (8R,9S,13S,14S)-3-[2-[[(2R,3S,4R)-5-(6-aminopurin-9-yl)-3,4-dihydroxy-tetrahydrofuran-2-yl]methoxy]ethoxy]-13-methyl-4-nitro-7,8,9,11,12,14,15,16-octahydro-6H-cyclopenta[a]phenanthren-17-one | C30 H36 N6 O8 | 26.581 | 37890672.57 | 608.26198 | 607.2547 |
|  | 3-Hydrazino-5-(trifluoromethyl)-1H-pyrazole | C4 H5 F3 N4 | 0.659 | 37698210.4 | 166.04655 | 225.06061 |
|  | 4-mercaptophenylboronic acid | C6 H7 B O2 S | 0.693 | 37527130.95 | 154.02539 | 213.03935 |
|  | (4E)-7-(Chlorocarbonyl)-2-pentyl-4-dodecenoic acid | C18 H31 Cl O3 | 20.476 | 37298948.64 | 330.19511 | 329.18784 |
|  | 1,1'-(1,3-dimethyl-2-oxoimidazolidine-4,5-diyl)bis(3-tert-butylurea) | C15 H30 N6 O3 | 14.927 | 37127566.7 | 342.23945 | 341.23218 |
|  | 2-(2-(Benzoyloxy)propoxy)propyl benzoate | C20 H22 O5 | 15.847 | 37026259.1 | 342.14564 | 341.13837 |
|  | Dithiocyano methane | C3 H2 N2 S2 | 0.626 | 36839191.68 | 129.96533 | 128.95805 |
|  | Neochlorogenic acid | C16 H18 O9 | 5.35 | 36057944.42 | 354.0941 | 353.08682 |
|  | N-{(1R,2S)-1-[(1R,2S)-2-(Butylcarbamoyl)cyclopropyl]-1,3-dihydroxy-2-propanyl}-Nalpha-{[(2-methyl-2-propanyl)oxy]carbonyl}-L-phenylalaninamide | C25 H39 N3 O6 | 20.236 | 34538386.54 | 477.28398 | 476.2767 |
|  | [Similar to: 4,5-Dicaffeoylquinic acid; ΔMass: 0.1571 Da] | C23 H41 Cl N6 O5 | 8.175 | 34523078.43 | 516.28383 | 515.27655 |
|  | 5-[6-(Dimethylamino)-9H-purin-9-yl]pentyl 6-[6-(dimethylamino)-9H-purin-9-yl]hexanoate | C25 H36 N10 O2 | 9.75 | 34514239.5 | 508.30205 | 507.29477 |
|  | Artemotil | C17 H28 O5 | 15.875 | 34205801.38 | 312.19285 | 311.18558 |
|  | terbufibrol | C20 H24 O5 | 20.35 | 34033618.28 | 344.16136 | 343.15408 |
|  | 4,6-Diamino-3-{[2-amino-5-(aminomethyl)-4-hydroxycyclohexyl]oxy}-2-hydroxycyclohexyl 2,6-diamino-2,6-dideoxyhexopyranoside | C19 H40 N6 O7 | 23.421 | 33753070.05 | 464.29725 | 463.28998 |
|  | 13(S)-HOTrE | C18 H30 O3 | 19.579 | 33339570.79 | 294.21864 | 293.21136 |
|  | SHPP | C13 H13 N O5 | 8.414 | 33264523.61 | 263.07863 | 262.07135 |
|  | (2'R,3R,4'R,4a'R,5S,8a'S)-5-(3-Furyl)-4'-hydroxy-4a',5'-bis(hydroxymethyl)-2'-methyl-3',4,4',4a',5,7',8',8a'-octahydro-2'H-spiro[furan-3,1'-naphthalen]-2-one | C20 H26 O6 | 13.942 | 32345249.77 | 362.17204 | 361.16476 |
|  | Phlinoside A | C35 H46 O20 | 8.042 | 32176497.36 | 786.25606 | 785.24878 |
|  | N-(2-{[4-(Ethylamino)-6-(isopropylamino)-1,3,5-triazin-2-yl]oxy}ethyl)-2,5-dimethylbenzenesulfonamide | C18 H28 N6 O3 S | 23.972 | 32143566.82 | 408.19328 | 407.186 |
|  | 2,3-Dihydroxypropyl 6-deoxy-6-sulfo-Î±-D-glucopyranoside | C9 H18 O10 S | 0.752 | 31650116.78 | 318.06093 | 317.05365 |
|  | (2E,2'E)-N,N'-1,3-Propanediylbis[2-cyano-3-(3,4-dihydroxy-5-nitrophenyl)acrylamide] | C23 H18 N6 O10 | 8.839 | 31375031.85 | 538.10702 | 537.09973 |
|  | valtrate | C22 H30 O8 | 12.106 | 31112803.96 | 422.19288 | 421.18561 |
|  | 3,3'-Diisopropyl-6,6'-dimethyl-2,2',5,5'-biphenyltetrol | C20 H26 O4 | 18.746 | 31050923.72 | 330.18217 | 329.1749 |
|  | Glycyl-L-prolyl-L-isoleucyl-L-prolyl-L-asparagine | C22 H36 N6 O7 | 11.696 | 30942484.35 | 496.26578 | 495.25854 |
|  | Î²-D-erythro-Pentofuranosyl chloride, 2-deoxy-, diacetate | C9 H13 Cl O5 | 2.036 | 30506289.8 | 236.04419 | 235.03691 |
|  | Sclareol | C23 H40 N6 O10 | 9.67 | 29458341.05 | 560.28157 | 559.27429 |
|  | (4E)-7-(Chlorocarbonyl)-2-pentyl-4-dodecenoic acid | C18 H31 Cl O3 | 20.701 | 29308509.55 | 330.19517 | 329.1879 |
|  | cetyl sulfate | C16 H34 O4 S | 26.355 | 28340703.05 | 322.21684 | 321.20956 |
|  | Decylubiquinone | C19 H30 O4 | 18.412 | 28123300.7 | 322.21351 | 321.20624 |
|  | 1-[3-{2-[(3-Aminopropyl)amino]-2-oxoethyl}-7,8-dimethyl-2,4-dioxo-3,4-dihydrobenzo[g]pteridin-10(2H)-yl]-1-deoxy-D-ribitol | C22 H30 N6 O7 | 14.395 | 27400645.94 | 490.21889 | 489.21161 |
|  | Ethyl (2Z)-3-[(2E)-2-(1,3-dimethyl-5-nitroso-2,6-dioxotetrahydro-4(1H)-pyrimidinylidene)hydrazino]-2-nitroacrylate | C11 H14 N6 O7 | 4.895 | 27375915.25 | 342.09407 | 341.08679 |
|  | 2-[2-(Tetradecyloxy)ethoxy]ethyl hydrogen sulfate | C18 H38 O6 S | 25.388 | 27287805.59 | 382.23778 | 381.2305 |
|  | 2,2,14,14-Tetramethyl-8-oxopentadecanedioic acid | C19 H34 O5 | 15.151 | 26883607.7 | 342.23948 | 341.23221 |
|  | 6-Hydroxy-1-(hydroxymethyl)-5-{2-[2-(hydroxymethyl)-1-pyrrolidinyl]-2-oxoethyl}-1,4a-dimethyldecahydro-2-naphthalenyl [3-(trifluoromethyl)phenyl]carbamate | C28 H39 F3 N2 O6 | 9.108 | 26441402.09 | 556.27876 | 555.27148 |
|  | N-Acetyl-L-alpha-aspartyl-L-seryl-L-phenylalanyl-L-alpha-aspartyl-L-glutamine | C27 H36 N6 O13 | 10.403 | 26436460.17 | 652.23472 | 697.23309 |
|  | {(1S,2S,3R,4R)-3-[(1S)-1-Acetamido-2-ethylbutyl]-4-carbamimidamido-2-hydroxycyclopentyl}phosphonic acid | C14 H29 N4 O5 P | 13.573 | 26355758.39 | 364.18742 | 363.18015 |
|  | 13(S)-HpOTrE | C18 H30 O4 | 17.287 | 26267625 | 310.21358 | 309.2063 |
|  | NP-020521 | C18 H32 O3 | 23.869 | 26206420.99 | 296.23405 | 295.22678 |
|  | Diisopropyl 3,3-dimethoxy-1,1-cyclobutanedicarboxylate | C14 H24 O6 | 9.178 | 26123637.04 | 288.15648 | 287.1492 |
|  | 1-{(2R)-2-[(tert-butoxycarbonyl)amino]-2-cyclohexylacetyl}-N-{(2S)-5-[(diaminomethylidene)amino]-1-oxopentan-2-yl}-L-prolinamide | C24 H42 N6 O5 | 14.22 | 25745575.36 | 494.32304 | 493.31577 |
|  | Ethyl N~2~-(3,4-dimethoxybenzyl)-N~5~-{[(2-methyl-2-propanyl)oxy]carbonyl}-L-ornithinate | C21 H34 N2 O6 | 23.549 | 25672561.1 | 410.24214 | 409.23486 |
|  | 13(S)-HOTrE | C18 H30 O3 | 19.416 | 25440540.5 | 294.21861 | 293.21133 |
|  | Ethyl (6-amino-4-{[5-(diethylamino)-2-pentanyl]amino}-5-nitro-2-pyridinyl)carbamate | C17 H30 N6 O4 | 11.404 | 24860839.87 | 382.23454 | 381.22726 |
|  | N-{5-[3-(4-Methoxyphenyl)-4-oxo-3,4-dihydro-2-quinazolinyl]-4-phenyl-1,3-thiazol-2-yl}benzamide | C31 H22 N4 O3 S | 10.981 | 24811681.13 | 530.14052 | 529.13324 |
|  | [Similar to: (2R,3R,4R,5R,6R)-2-({[(2S,3R,4R)-3,4-dihydroxy-4-(hydroxymethyl)oxolan-2-yl]oxy}methyl)-6-[2-(3,4-dimethoxyphenyl)ethoxy]-5-hydroxy-4-{[(2S,3R,4R,5R,6S)-3,4,5-trihydroxy-6-methyloxan-2-yl]oxy}oxan-3-yl (2E)-3-(3,4-dihydroxyphenyl)prop-2-enoate; ΔMass: 30.0082 Da] | C38 H44 N10 O7 P2 | 9.97 | 24688972.34 | 814.28714 | 813.27991 |
|  | Neochlorogenic acid | C16 H18 O9 | 3.028 | 24358596.99 | 354.09422 | 353.08694 |
|  | {4a,6-Dihydroxy-1-[(3-methylbutanoyl)oxy]-4a,5,6,7a-tetrahydro-1H-spiro[cyclopenta[c]pyran-7,2'-oxiran]-4-yl}methyl 3-methyl-2-[(3-methylbutanoyl)oxy]butanoate | C25 H38 O10 | 13.705 | 23990982.93 | 498.24504 | 497.23776 |
|  | 3,3'-[7,12-Bis(1,2-dihydroxyethyl)-3,8,13,17-tetramethyl-2,18-porphyrindiyl]dipropanoic acid | C34 H38 N4 O8 | 9.431 | 23065588.18 | 630.27156 | 629.26428 |
|  | 6-{1-[2-(Dipropylamino)-2-oxoethyl]-2,4-dioxo-1,4-dihydrothieno[3,2-d]pyrimidin-3(2H)-yl}-N-phenylhexanamide | C26 H34 N4 O4 S | 9.312 | 22650124.01 | 498.22993 | 497.22266 |
|  | 4,5-Dicaffeoylquinic acid | C25 H24 O12 | 10.358 | 22419957.4 | 516.12526 | 515.11798 |
|  | 2-(3-CARBOXYPROPIONYL)-6-HYDROXY-CYCLOHEXA-2,4-DIENE CARBOXYLIC ACID | C11 H12 O6 | 4.985 | 22353707 | 240.06244 | 239.05516 |
|  | [Similar to: Chlorogenic acid; ΔMass: -162.0694 Da] | C10 H10 P2 | 0.685 | 21824296.42 | 192.02563 | 191.01836 |
|  | [Similar to: Sorbitan monooleate; ΔMass: 81.9802 Da] | C19 H44 N8 O4 P2 | 23.832 | 21763081.15 | 510.29399 | 509.28671 |
|  | 1-[4,5-Dihydroxy-6-(hydroxymethyl)-3-[(E)-3-(4-hydroxyphenyl)prop-2-enoyl]oxyoxan-2-yl]oxy-7-hydroxy-7-methyl-4a,5,6,7a-tetrahydro-1H-cyclopenta[c]pyran-4-carboxylic acid | C25 H30 O12 | 10.122 | 21714045.49 | 522.17213 | 567.17029 |
|  | N-(Dipropoxyphosphoryl)-L-alanyl-L-alanyl-L-alanyl-L-alanine | C18 H35 N4 O8 P | 11.887 | 21688320.85 | 466.21876 | 465.21149 |
|  | TC5470000 | C12 H11 O4 P | 9.183 | 21642616.18 | 250.03857 | 249.0313 |
|  | 3-O-Ethylascorbic acid | C8 H12 O6 | 0.675 | 21388211.36 | 204.06251 | 407.11819 |
|  | DOG | C19 H36 O5 | 17.254 | 21172890.43 | 344.25529 | 343.24802 |
|  | Ethyl (2Z)-3-[(2E)-2-(1,3-dimethyl-5-nitroso-2,6-dioxotetrahydro-4(1H)-pyrimidinylidene)hydrazino]-2-nitroacrylate | C11 H14 N6 O7 | 4.292 | 20848003.11 | 342.09398 | 341.0867 |
|  | [Similar to: Glycitein; ΔMass: 208.0570 Da] | C21 H27 N4 O4 P3 | 11.554 | 20832617.32 | 492.1255 | 491.11823 |
|  | 1,1'-(2-Chloro-1,3-propanediyl)bis(3-cyclohexyl-1-nitrosourea) | C17 H29 Cl N6 O4 | 12.308 | 20817517.06 | 416.19523 | 415.18796 |
|  | Diisopropyl 3-hydroxy-1,1-cyclobutanedicarboxylate | C12 H20 O5 | 9.507 | 20633853.19 | 244.13008 | 243.1228 |
|  | Ethyl 2-(3,5-dimethyl-1H-pyrazol-1-yl)-4-[2-(2-methyl-6-phenyl-4-pyrimidinyl)hydrazino]-5-pyrimidinecarboxylate | C23 H24 N8 O2 | 17.287 | 20527536.37 | 444.20348 | 489.20166 |
|  | [Similar to: Gluconic acid; ΔMass: -0.0225 Da] | C3 H9 N4 O4 P | 0.689 | 20298359.88 | 196.03584 | 177.0179 |
|  | [Similar to: NP-017873; ΔMass: -392.1952 Da] | C16 H27 Cl N6 O3 | 12.754 | 20217423.39 | 386.18233 | 385.17505 |
|  | NP-005870 | C20 H26 O5 | 13.052 | 20209876.4 | 346.17695 | 345.16968 |
|  | {(1S,2S,3R,4R)-3-[(1S)-1-Acetamido-2-ethylbutyl]-4-carbamimidamido-2-hydroxycyclopentyl}phosphonic acid | C14 H29 N4 O5 P | 13.376 | 20005653.59 | 364.18756 | 363.1803 |
|  | Prostaglandin E2 | C20 H32 O5 | 13.468 | 19948813.13 | 352.22389 | 351.21661 |
|  | N-Acetyl-L-alpha-aspartyl-L-seryl-L-phenylalanyl-L-alpha-aspartyl-L-glutamine | C27 H36 N6 O13 | 10.954 | 19929185.93 | 652.23463 | 651.22729 |
|  | NP-005870 | C20 H26 O5 | 20.761 | 19686911.04 | 346.17674 | 345.16946 |
|  | (±)9-HpODE | C18 H32 O4 | 18.459 | 19634186.63 | 312.2292 | 311.22192 |
|  | (1R,2S,3S,4R,6S)-4,6-Diamino-2-[(3-{2-[(3-aminopropyl)amino]ethyl}-3-deoxy-beta-D-ribofuranosyl)oxy]-3-hydroxycyclohexyl 2,6-diamino-2,6-dideoxy-alpha-D-galactopyranoside | C22 H46 N6 O9 | 22.93 | 19458757.01 | 538.33363 | 537.32635 |
|  | Juniperic acid | C16 H32 O3 | 24.054 | 19411977.16 | 272.23424 | 271.22696 |
|  | Alanylalanylvalyl-N-(4-nitrophenyl)alaninamide | C20 H30 N6 O6 | 13.046 | 19078337.01 | 450.22417 | 449.21689 |
|  | 3,3'-Diisopropyl-6,6'-dimethyl-2,2',5,5'-biphenyltetrol | C20 H26 O4 | 18.866 | 18665308.31 | 330.18226 | 329.17499 |
|  | PEGUNIGALSIDASE ALFA | C24 H44 N6 O9 | 19.707 | 18614966.84 | 560.31795 | 559.31067 |
|  | (2S)-1-(Methylamino)-1-oxo-2-propanyl 2-({(2S)-1-[(2S)-2-{[(2S)-2-acetamidopropanoyl]amino}propanoyl]-2-pyrrolidinyl}carbonyl)-1-methylhydrazinecarboxylate (non-preferred name) | C19 H32 N6 O7 | 11.294 | 18543044.66 | 456.23479 | 455.22751 |
|  | 6,10,10b-Trihydroxy-3,4a,7,7,10a-pentamethyl-1-oxo-3-vinyldodecahydro-1H-benzo[f]chromen-5-yl acetate | C22 H34 O7 | 18.245 | 18533706.86 | 410.22923 | 409.22195 |
|  | Methyl 1-[3-({[5-carbamoyl-4-ethyl-6-(4-nitrophenyl)-2-oxo-3,6-dihydro-1(2H)-pyrimidinyl]carbonyl}amino)propyl]-4-phenyl-4-piperidinecarboxylate | C30 H36 N6 O7 | 27.146 | 18105877.28 | 592.26674 | 591.25946 |
|  | (4-Amino-7-isopropyl-7H-pyrrolo[2,3-d]pyrimidin-5-yl)(6-{[2-(3-pyridinyl)ethyl]amino}-2-pyrazinyl)methanone | C21 H22 N8 O | 15.117 | 17720514.51 | 402.19289 | 437.16223 |
|  | 2,4-dihydroxyheptadec-16-ynyl acetate | C19 H34 O4 | 19.128 | 17714553.76 | 326.24473 | 325.23746 |
|  | Glycyl-L-prolyl-L-isoleucyl-L-prolyl-L-asparagine | C22 H36 N6 O7 | 13.156 | 17574683.22 | 496.26585 | 495.25858 |
|  | 3-Pyridine(~14~C)carboxamide | C5 [14]C H6 N2 O | 1.794 | 17526078.06 | 124.05083 | 123.04355 |
|  | Phlinoside A | C35 H46 O20 | 8.562 | 17440475.1 | 786.2563 | 785.24902 |
|  | Anthra[1,2-c][1,2,5]oxadiazole-6,11-dione | C14 H6 N2 O3 | 9.376 | 17203318.64 | 250.03856 | 249.03128 |
|  | 19(R)-Hydroxy prostaglandin F2α | C20 H34 O6 | 10.057 | 17136816.19 | 370.23445 | 369.22717 |
|  | 4-({5-[(2-aminoethyl)amino]-2,4-dinitrophenyl}amino)-TEMPO | C17 H28 N6 O5 | 12.926 | 17022431.5 | 396.2111 | 395.20383 |
|  | 1,2,3,4-Tetrahydro-7-hydroxy-1-(4-hydroxy-3-methoxyphenyl)-6-methoxy-2,3-naphthalenedimethanol | C20 H24 O6 | 11.593 | 16773680.32 | 360.15626 | 359.14899 |
|  | [Similar to: (3beta,5xi,9xi,22beta)-22,24-Dihydroxyolean-12-en-3-yl 6-deoxy-alpha-L-mannopyranosyl-(1-2)-beta-D-galactopyranuronosyl-(1-2)-beta-D-glucopyranosiduronic acid; ΔMass: -304.3723 Da] | C35 H21 N6 O6 P | 8.16 | 16659864.38 | 652.12581 | 651.11853 |
|  | 2,2,14,14-Tetramethyl-8-oxopentadecanedioic acid | C19 H34 O5 | 15.94 | 16573198.65 | 342.23964 | 341.23236 |
|  | 5-[6-(Dimethylamino)-9H-purin-9-yl]pentyl 6-[6-(dimethylamino)-9H-purin-9-yl]hexanoate | C25 H36 N10 O2 | 12.606 | 16519352.74 | 508.30192 | 507.29465 |
|  | (2E,2'E)-N,N'-1,3-Propanediylbis[2-cyano-3-(3,4-dihydroxy-5-nitrophenyl)acrylamide] | C23 H18 N6 O10 | 9.419 | 16427103.8 | 538.10713 | 537.09973 |
|  | HP8757000 | C14 H10 O5 | 11.429 | 16276530.32 | 258.05198 | 257.04471 |
|  | MFCD09869739 | C14 H22 O5 | 10.981 | 16239444.77 | 270.1458 | 269.13852 |
|  | L-Seryl-L-threonyl-L-leucyl-L-asparaginyl-L-phenylalanine | C26 H40 N6 O9 | 17.304 | 16145824.84 | 580.28651 | 579.27924 |
|  | 4-({5-[(2-aminoethyl)amino]-2,4-dinitrophenyl}amino)-TEMPO | C17 H28 N6 O5 | 13.425 | 15777072.28 | 396.21098 | 395.2037 |
|  | Methyl 2-[5-{[(trans-4-aminocyclohexyl)methyl]carbamoyl}-1,3-dioxo-5,8-dihydro-1H-[1,2,4]triazolo[1,2-a]pyridazin-2(3H)-yl]-3-(cyclohexylamino)propanoate | C24 H38 N6 O5 | 12.124 | 15516941.52 | 490.29185 | 489.28458 |
|  | Quercetin-3β-D-glucoside | C21 H20 O12 | 8.16 | 15380111.45 | 464.09434 | 463.08707 |
|  | 2,2'-[(6-Chloro-2,4-pyrimidinediyl)di-4,1-piperazinediyl]diethanol | C16 H27 Cl N6 O2 | 16.811 | 15335211.73 | 370.18742 | 369.18015 |
|  | 4-({5-[(2-aminoethyl)amino]-2,4-dinitrophenyl}amino)-TEMPO | C17 H28 N6 O5 | 14.327 | 15255154.94 | 396.21092 | 395.20364 |
|  | (2R,3R,4S,5R)-2-(6-Amino-2-{[(2S)-1-(4-methoxyphenyl)-2-propanyl]amino}-9H-purin-9-yl)-5-(2-ethyl-2H-tetrazol-5-yl)tetrahydro-3,4-furandiol | C22 H28 N10 O4 | 12.842 | 14857694.66 | 496.22951 | 495.22223 |
|  | 1,3-Dioxo-2-(2-pyrimidinyl)-5-isoindolinecarboxylic acid | C13 H7 N3 O4 | 15.463 | 14682351.28 | 269.04356 | 268.03629 |
|  | Glaurin | C16 H32 O4 | 14.029 | 14573094.84 | 288.22923 | 287.22195 |
|  | 1-Cyano-2-oxo-1-propanediazonium | C4 H4 N3 O | 1.385 | 14444777.79 | 110.03513 | 109.02785 |
|  | Tricin | C17 H14 O7 | 6.478 | 14268433.8 | 330.07326 | 329.06598 |
|  | 1-O-[(5beta,6beta,8alpha,9beta,10alpha,11beta,13alpha)-6,11-Dihydroxy-15,18-dioxokaur-16-en-18-yl]-beta-D-glucopyranose | C26 H38 O10 | 9.442 | 14060026.28 | 510.24495 | 509.23767 |
|  | Diethyl diallylmalonate | C13 H20 O4 | 14.371 | 13896998.57 | 240.13508 | 239.12781 |
|  | 3-{[(4R,5S,6S,7R)-4,7-Dibenzyl-5,6-dihydroxy-3-(3-methoxybenzyl)-2-oxo-1,3-diazepan-1-yl]methyl}-N-(5-methyl-2-pyridinyl)benzamide | C41 H42 N4 O5 | 13.942 | 13788431.65 | 670.31819 | 669.31091 |
|  | 5'-O-[(2E,6S,7R)-6,7-Dihydroxy-3,7,11-trimethyl-2,10-dodecadien-1-yl]-5,6-dihydrouridine | C24 H40 N2 O8 | 22.463 | 13775539.33 | 484.27888 | 483.27161 |
|  | 1,1'-(1,3-dimethyl-2-oxoimidazolidine-4,5-diyl)bis(3-tert-butylurea) | C15 H30 N6 O3 | 16.599 | 13761497.16 | 342.23961 | 341.23233 |
|  | NP-005870 | C20 H26 O5 | 12.578 | 13677764.9 | 346.17692 | 345.16965 |
|  | L-Tyrosyl-L-valyl-L-lysyl-L-alanyl-L-alanine | C26 H42 N6 O7 | 10.929 | 13606417.98 | 550.31257 | 549.3053 |
|  | 4,4'-Sulfonylbis(2-allylphenol) | C18 H18 O4 S | 0.713 | 13447693.47 | 330.09398 | 365.06339 |
|  | N,N'-Bis(2,4-dinitrophenyl)-1,6-hexanediamine | C18 H20 N6 O8 | 8.802 | 13376123.46 | 448.13548 | 447.1282 |
|  | 5-(3,5-Dinitrophenyl)-2H-tetrazole | C7 H4 N6 O4 | 1.791 | 13374876.69 | 236.02865 | 235.02138 |
|  | Dehydrodiisoeugenol | C20 H22 O4 | 19.503 | 12859720.69 | 326.15065 | 325.14337 |
|  | 15-Hydroxypentadecanoic acid | C15 H30 O3 | 22.324 | 12801622.79 | 258.21858 | 257.2113 |
|  | 20β-Dihydroprednisone | C21 H28 O5 | 16.085 | 12511192.91 | 360.19276 | 359.18549 |
|  | [Similar to: (2R,3R,4R,5R,6R)-2-({[(2S,3R,4R)-3,4-dihydroxy-4-(hydroxymethyl)oxolan-2-yl]oxy}methyl)-6-[2-(3,4-dimethoxyphenyl)ethoxy]-5-hydroxy-4-{[(2S,3R,4R,5R,6S)-3,4,5-trihydroxy-6-methyloxan-2-yl]oxy}oxan-3-yl (2E)-3-(3,4-dihydroxyphenyl)prop-2-enoate; ΔMass: -146.0599 Da] | C26 H34 N6 O13 | 9.225 | 12463845.17 | 638.2191 | 637.21179 |
|  | 1-[(Z)-(2,4-Dinitrophenoxy)-NNO-azoxy]-3-piperidinecarboxamide | C12 H14 N6 O7 | 0.977 | 12300992.81 | 354.09401 | 353.08673 |
|  | Diisopropyl 3-hydroxy-1,1-cyclobutanedicarboxylate | C12 H20 O5 | 10.161 | 12278937.38 | 244.13016 | 243.12288 |
|  | Phenyl N,N'-di-4-morpholinylphosphorodiamidate | C14 H23 N4 O4 P | 14.388 | 12159000.45 | 342.14567 | 341.1384 |
|  | Diisopropyl 3-hydroxy-1,1-cyclobutanedicarboxylate | C12 H20 O5 | 9.596 | 12128125.98 | 244.13013 | 243.12285 |
|  | O-2-Decanyl O,O-bis(2-ethoxyethyl) phosphorothioate | C18 H39 O5 P S | 15.391 | 12086458.64 | 398.22664 | 397.21936 |
|  | L-Prolyl-L-glutaminyl-L-isoleucyl-L-threonyl-L-leucine | C26 H46 N6 O8 | 8.18 | 12052413.61 | 570.33876 | 569.33148 |
|  | [Similar to: NP-007494; ΔMass: -424.1743 Da] | C5 H15 N4 O6 P | 1.396 | 11996058.34 | 258.07298 | 257.0657 |
|  | Hispidulin | C16 H12 O6 | 12.922 | 11878924.41 | 300.06245 | 299.05518 |
|  | Euxanthone | C13 H8 O4 | 13.218 | 11873478.52 | 228.04123 | 227.03395 |
|  | BIS-HEMA IPDI | C24 H38 N2 O8 | 21.737 | 11868215.48 | 482.26301 | 481.25574 |
|  | N2,N4,N6-Tripivaloyloxymethyl-N2,N4,N6-trimethylmelamine | C24 H42 N6 O6 | 11.907 | 11758378.8 | 510.31767 | 509.31039 |
|  | Methyl 2-[5-{[(trans-4-aminocyclohexyl)methyl]carbamoyl}-1,3-dioxo-5,8-dihydro-1H-[1,2,4]triazolo[1,2-a]pyridazin-2(3H)-yl]-3-(cyclohexylamino)propanoate | C24 H38 N6 O5 | 12.896 | 11661223.13 | 490.29173 | 489.28445 |
|  | MFCD00083370 | C22 H44 O3 | 28.463 | 11622493.18 | 356.32789 | 355.32062 |
|  | Bis(2-methyl-2-propanyl) 4,4'-(1H-imidazole-4,5-diyldicarbonyl)di(1-piperazinecarboxylate) | C23 H36 N6 O6 | 19.448 | 11534269.18 | 492.27083 | 491.26355 |
|  | (2E,2'E)-N,N'-1,3-Propanediylbis[2-cyano-3-(3,4-dihydroxy-5-nitrophenyl)acrylamide] | C23 H18 N6 O10 | 8.703 | 11524079.72 | 538.10695 | 537.09967 |
|  | N-[(1S)-5-Amino-1-{5-[(1S)-1-amino-2-hydroxyethyl]-1,3,4-oxadiazol-2-yl}pentyl]-4-piperidinecarboxamide | C15 H28 N6 O3 | 25.034 | 11445343.16 | 340.2213 | 339.21402 |
|  | NP-005870 | C20 H26 O5 | 12.772 | 11441879.89 | 346.17705 | 345.16977 |
|  | 1-(2,5-Dideoxy-5-{5-[(2,4-dioxo-3,4-dihydro-1(2H)-pyrimidinyl)methyl]-1H-1,2,3-triazol-1-yl}pentofuranosyl)-5-methyl-2,4(1H,3H)-pyrimidinedione | C17 H19 N7 O6 | 9.674 | 11414512.5 | 417.14116 | 416.13388 |
|  | Ethyl 1-{3-[5-(4-morpholinylmethyl)-1H-tetrazol-1-yl]propanoyl}-2-piperidinecarboxylate | C17 H28 N6 O4 | 12.697 | 11399285.88 | 380.21879 | 379.21152 |
|  | (2R,4S)-2-{(1R)-2-(Benzylamino)-2-oxo-1-[(phenylacetyl)amino]ethyl}-5,5-dimethyl-N-[(2S)-1-phenyl-2-propanyl]-1,3-thiazolidine-4-carboxamide | C32 H38 N4 O3 S | 10.953 | 11109528.2 | 558.2657 | 557.25842 |
|  | N-{4-[(1-{2,6-Dideoxy-4-O-[4,6-dideoxy-4-(dimethylamino)hexopyranosyl]hexopyranosyl}-2-oxo-1,2-dihydro-4-pyrimidinyl)carbamoyl]phenyl}-2-methylserinamide | C29 H42 N6 O10 | 9.108 | 11088902.61 | 634.29481 | 633.28754 |
|  | Dehydrodiisoeugenol | C20 H22 O4 | 20.578 | 10938553.68 | 326.15074 | 325.14346 |
|  | Tetradecyl 4-chloro-4-oxobutanoate | C18 H33 Cl O3 | 21.836 | 10868244.39 | 332.21092 | 331.20364 |
|  | 1,1'-(1,6-Hexanediyl)bis[3-(2-hydroxyethyl)-1-nitrosourea] | C12 H24 N6 O6 | 8.833 | 10846635.5 | 348.17738 | 347.1701 |
|  | 1,6-Hexanediol diacrylate | C12 H18 O4 | 6.769 | 10792592.32 | 226.11944 | 225.11217 |
|  | [Similar to: 2-(3,4-Dihydroxyphenyl)ethyl 3-O-(6-deoxy-β-L-mannopyranosyl)-6-O-[(2E)-3-(3,4-dihydroxyphenyl)-2-propenoyl]-β-D-glucopyranoside; ΔMass: -146.0592 Da] | C20 H18 N10 O5 | 8.436 | 10765071.07 | 478.14622 | 477.13895 |
|  | 1,2,3,4-Tetrahydro-7-hydroxy-1-(4-hydroxy-3-methoxyphenyl)-6-methoxy-2,3-naphthalenedimethanol | C20 H24 O6 | 11.808 | 10625516.15 | 360.15645 | 359.14917 |
|  | 2-Furyl(5-hydroxy-1-benzofuran-3-yl)methanone | C13 H8 O4 | 11.572 | 10605493.17 | 228.04117 | 227.03389 |
|  | N-(3-{Benzyl[(diphenylmethyl)carbamoyl]amino}-2-hydroxypropyl)-N-[2-(4-morpholinyl)ethyl]-1-phenylmethanesulfonamide | C37 H44 N4 O5 S | 12.457 | 10534899.04 | 656.3025 | 655.29523 |
|  | 19(R)-Hydroxy prostaglandin A2 | C20 H30 O5 | 14.311 | 10484246.6 | 350.2082 | 349.20093 |
|  | (±)9-HpODE | C18 H32 O4 | 18.054 | 10212198.09 | 312.22938 | 311.22211 |
|  | (.+/-.)-Usnic acid | C18 H16 O7 | 10.304 | 10153493.21 | 344.0887 | 343.08142 |
|  | (2S,3S,4R,5R,6S)-2-[Chloro(hydroxy)methyl]-6-{[(4aR)-7-hydroxy-7-(hydroxymethyl)-1,4a,5,6,7,7a-hexahydrocyclopenta[c]pyran-1-yl]oxy}tetrahydro-2H-pyran-2,3,4,5-tetrol | C15 H23 Cl O10 | 1.569 | 10118822.62 | 398.09672 | 397.08945 |
|  | MFCD09869739 | C14 H22 O5 | 11.517 | 10051331.03 | 270.14598 | 269.1387 |
|  | N-Acetyl-L-alpha-glutamylglycyl-L-threonyl-L-tyrosylglycinamide | C24 H34 N6 O10 | 13.331 | 10018443.05 | 566.23244 | 565.22516 |
|  | ganoderic acid E | C30 H40 O7 | 16.528 | 9958985.472 | 512.27601 | 511.26874 |
|  | ZK118182 | C21 H33 Cl O5 | 18.246 | 9863218.061 | 400.20039 | 399.19312 |
|  | 12-(2-Cyanoethyl)-6,7,12,13-tetrahydro-13-methyl-5-oxo-5H-indolo[2,3-a]pyrrolo[3,4-c]carbazole | C24 H18 N4 O | 25.04 | 9780115.62 | 378.1487 | 377.14142 |
|  | [Similar to: Chlorogenic acid; ΔMass: -162.1162 Da] | C3 H6 N4 P2 S | 0.781 | 9659129.551 | 191.9789 | 190.97162 |
|  | 3,3'-[7,12-Bis(1,2-dihydroxyethyl)-3,8,13,17-tetramethyl-2,18-porphyrindiyl]dipropanoic acid | C34 H38 N4 O8 | 9.189 | 9623074.367 | 630.27168 | 629.2644 |
|  | 1,2,3,4-Tetrahydro-7-hydroxy-1-(4-hydroxy-3-methoxyphenyl)-6-methoxy-2,3-naphthalenedimethanol | C20 H24 O6 | 12.16 | 9553633.768 | 360.15635 | 359.14908 |
|  | DOG | C19 H36 O5 | 17.094 | 9373293.059 | 344.2552 | 343.24792 |
|  | 2-(3,4-Dihydroxyphenyl)ethyl 3-O-(6-deoxy-β-L-mannopyranosyl)-6-O-[(2E)-3-(3,4-dihydroxyphenyl)-2-propenoyl]-β-D-glucopyranoside | C29 H36 O15 | 9.128 | 9351274.811 | 624.20369 | 623.19641 |
|  | 1,9-NONANEDIOL DIACRYLATE | C15 H24 O4 | 16.353 | 9345256.416 | 268.16661 | 267.15933 |
|  | [Similar to: NP-021293; ΔMass: 21.8561 Da] | C16 H40 N4 O7 P2 S | 12.309 | 9127758.641 | 494.21141 | 493.20413 |
|  | Glycyl-L-alanyl-L-alanylglycyl-L-alanyl-L-alanine | C16 H28 N6 O7 | 8.997 | 9115473.452 | 416.20354 | 415.19626 |
|  | Rutin | C27 H30 O16 | 7.814 | 8941555.221 | 610.15169 | 609.14441 |
|  | 2-(Dibenzylamino)-3-phenylpropyl 4-(2-oxo-2,3-dihydro-1H-benzimidazol-1-yl)-1-piperidinecarboxylate | C36 H38 N4 O3 | 13.715 | 8920870.31 | 574.29707 | 573.28979 |
|  | L-Isoleucyl-L-isoleucyl-L-alanyl-L-isoleucyl-L-threonyl-3-cyclohexyl-L-alanine | C34 H62 N6 O8 | 13.573 | 8871098.632 | 682.46083 | 681.45355 |
|  | (2S)-2-(2-{[(1S)-3-(5-{(Z)-[(5S)-5-(3-Carbamimidamidopropyl)-3,6-dioxo-2-piperazinylidene]methyl}-2-hydroxyphenoxy)-1-carboxypropyl]amino}-2-oxoethyl)-2-hydroxysuccinic acid | C25 H32 N6 O12 | 8.979 | 8761196.958 | 608.20875 | 607.20148 |
|  | L-Leucyl-L-prolyl-L-glutaminyl-L-threonyl-L-alanine | C23 H40 N6 O8 | 20.715 | 8736377.553 | 528.29164 | 527.28436 |
|  | 2-[(4-Ethyl-5-{4-[(4-methyl-1-piperazinyl)sulfonyl]phenyl}-4H-1,2,4-triazol-3-yl)sulfanyl]-1-(2-methyl-1-piperidinyl)ethanone | C23 H34 N6 O3 S2 | 12.662 | 8540053.184 | 506.21358 | 505.2063 |
|  | (4-Fluorophenyl)(trimethyl)silane | C9 H13 F Si | 6.887 | 8489941.187 | 168.07722 | 167.06995 |
|  | 3,3'-(2-Oxido-1,2,5-oxadiazole-3,4-diyl)bis(5-nitroaniline) | C14 H10 N6 O6 | 1.385 | 8407183.377 | 358.06538 | 357.05811 |
|  | Selodenoson | C17 H24 N6 O4 | 15.113 | 8356355.625 | 376.18504 | 375.17776 |
|  | terbufibrol | C20 H24 O5 | 19.261 | 8294538.692 | 344.16127 | 343.15399 |
|  | 1-(Butylsulfanyl)borepane | C10 H21 B S | 13.278 | 8272547.606 | 184.14496 | 183.13768 |
|  | Alanylalanylvalyl-N-(4-nitrophenyl)alaninamide | C20 H30 N6 O6 | 11.796 | 8223638.201 | 450.22224 | 449.21497 |
|  | 2-{1,3-Dimethyl-8-[(3-methyl-1-piperidinyl)methyl]-2,6-dioxo-1,2,3,6-tetrahydro-7H-purin-7-yl}acetamide | C16 H24 N6 O3 | 18.075 | 7972260.347 | 348.19264 | 347.18536 |
|  | Neochlorogenic acid | C16 H18 O9 | 8.842 | 7900948.555 | 354.0934 | 353.08612 |
|  | 2,2'-(Nitrosoimino)bis(N'-phenylacetohydrazide) | C16 H18 N6 O3 | 14.709 | 7893793.118 | 342.14564 | 341.13837 |
|  | 1,6-Hexanediol diacrylate | C12 H18 O4 | 11.458 | 7805011.929 | 226.11946 | 225.11218 |
|  | N-(6-Amino-1-isobutyl-2,4-dioxo-1,2,3,4-tetrahydro-5-pyrimidinyl)-2-[4-(4-hydroxyphenyl)-1-piperazinyl]-N-isobutylacetamide | C24 H36 N6 O4 | 15.369 | 7667706.039 | 472.28087 | 471.27359 |
|  | 17-hydroxy-3,6,9,12,15-pentaoxaheptadecyl acrylate | C15 H28 O8 | 10.822 | 7614533.345 | 336.17753 | 335.17026 |
|  | N-[(2S)-2-Aminobutanoyl]-L-threonyl-L-asparaginyl-N-[(1S)-1-carboxypropyl]-L-tyrosinamide | C25 H38 N6 O9 | 14.624 | 7491739.576 | 566.27095 | 565.26367 |
|  | [Similar to: 19(R)-hydroxy Prostaglandin F1?; ΔMass: -5.0506 Da] | C12 H29 N7 O4 S | 12.752 | 7454377.797 | 367.20061 | 366.19333 |
|  | [Similar to: 3,3'-Diisopropyl-6,6'-dimethyl-2,2',5,5'-biphenyltetrol; ΔMass: 324.1043 Da] | C28 H42 N6 O12 | 12.812 | 7400407.109 | 654.28737 | 653.28009 |
|  | 4beta-Phorbol | C20 H28 O6 | 14.256 | 7284582.554 | 364.18767 | 363.18039 |
|  | (Dimethylamino)[(2,5-dioxo-1-pyrrolidinyl)oxy]-N,N-dimethylmethaniminium | C9 H16 N3 O3 | 12.035 | 7262335.467 | 214.11937 | 213.11209 |
|  | Rutin | C27 H30 O16 | 7.927 | 7172929.157 | 610.15169 | 609.14441 |
|  | N,N'-(1,4-Piperazinediyldi-3,1-propanediyl)bis[2-(3,4,5-trimethoxyphenoxy)acetamide] | C32 H48 N4 O10 | 19.868 | 7120217.511 | 648.3343 | 647.32703 |
|  | (E)-N-(4-Amino-3-{[3-amino-6-(1-aminoethyl)tetrahydro-2H-pyran-2-yl]oxy}-2,5-dihydroxy-6-methoxycyclohexyl)-N~2~-(aminomethylene)-N-methylglycinamide | C18 H36 N6 O6 | 15.746 | 7012756.233 | 432.27092 | 431.26364 |
|  | [Similar to: NP-014604; ΔMass: 76.0583 Da] | C21 H44 Cl N4 O5 P | 9.48 | 6872856.907 | 498.27348 | 497.2662 |
|  | Quercetin-3β-D-glucoside | C21 H20 O12 | 8.036 | 6857173.631 | 464.09434 | 463.08707 |
|  | (2S,3R,4S,5R,8R,10R,11R,12R,13R,14R)-13-[(2,6-Dideoxy-3-C-methyl-3-O-methyl-alpha-D-lyxo-hexopyranosyl)oxy]-2-ethyl-3,4,10-trihydroxy-3,5,6,8,10,12,14-heptamethyl-15-oxo-1-oxa-6-azacyclopentadecan-11- yl 3,4,6-trideoxy-3-[(4-{1-[5-(hydroxyamino)-5-oxopentyl]-1H-1,2,3-triazol-4-yl}benzyl)(methyl)amino]-beta-D-arabino-hexopyranoside | C51 H86 N6 O14 | 8.179 | 6524584.422 | 1006.62031 | 1005.61304 |
|  | N-(1,3-Benzodioxol-5-ylmethyl)-3-[(3-nitro-1H-pyrazol-1-yl)methyl]-1,2,4-oxadiazole-5-carboxamide | C15 H12 N6 O6 | 8.118 | 6471502.436 | 372.08348 | 371.0762 |
|  | (3R,4aR,10S,10bS)-6,10,10b-Trihydroxy-3,4a,7,7,10a-pentamethyl-1-oxo-3-vinyldodecahydro-1H-benzo[f]chromen-5-yl 4-(1,3-dimethyl-2,6-dioxo-1,2,3,6-tetrahydro-7H-purin-7-yl)butanoate | C31 H44 N4 O9 | 14.707 | 6463291.814 | 616.30775 | 615.30048 |
|  | Rubiadin | C15 H10 O4 | 13.135 | 6396810.689 | 254.05696 | 253.04968 |
|  | [Similar to: 9-Nitrooleate; ΔMass: 182.0409 Da] | C18 H40 N9 O6 P | 21.662 | 6229842.727 | 509.28187 | 508.2746 |
|  | 1-{5-[(4,5-Diphenyl-1H-imidazol-2-yl)sulfanyl]pentyl}-3,5-dimethyl-1H-pyrazole | C25 H28 N4 S | 9.51 | 6138609.388 | 416.20363 | 415.19635 |
|  | N~2~-Acetyl-D-arginyl-L-arginyl-3-(2-naphthyl)-L-alaninamide | C27 H40 N10 O4 | 9.184 | 6068046.388 | 568.32319 | 567.31592 |
|  | [Similar to: NP-017873; ΔMass: -366.2135 Da] | C22 H33 Cl O S2 | 12.104 | 6005431.073 | 412.16411 | 411.15683 |
|  | WJ2510000 | C29 H44 O8 | 13.778 | 5943144.963 | 520.3022 | 519.29492 |
|  | MFCD04020245 | C16 H24 N6 O4 | 14.46 | 5933125.45 | 364.18757 | 363.1803 |
|  | DESNICOTINYL INDINAVIR | C30 H42 N4 O4 | 10.467 | 5885277.703 | 522.31807 | 521.31079 |
|  | Corchorifatty acid F | C18 H32 O5 | 16.376 | 5743664.236 | 328.22404 | 327.21677 |
|  | {(1S,2S,3R,4R)-3-[(1S)-1-Acetamido-2-ethylbutyl]-4-carbamimidamido-2-hydroxycyclopentyl}phosphonic acid | C14 H29 N4 O5 P | 15.681 | 5688990.611 | 364.18788 | 363.1806 |
|  | 8-Hydroxy-1,4-dioxo-1,4-dihydro-2-naphthalenyl 2,3,4,6-tetra-O-acetyl-beta-D-glucopyranoside | C24 H24 O13 | 13.422 | 5480462.566 | 520.12013 | 519.11285 |
|  | 1-Ethyl-4-(4-oxido-2,6-diphenyl-4H-1,4-oxaphosphinin-4-yl)piperazine | C22 H25 N2 O2 P | 26.613 | 5414945.819 | 380.16441 | 379.15714 |
|  | N-Acetyl-L-leucyl-L-alpha-aspartyl-L-seryl-L-seryl-L-leucinamide | C24 H42 N6 O10 | 13.873 | 5258008.198 | 574.29707 | 573.28979 |
|  | N-Acetyl-L-leucyl-L-alanyl-L-threonyl-L-seryl-L-leucinamide | C24 H44 N6 O8 | 9.943 | 5237998.834 | 544.32319 | 543.31592 |
|  | (1-Amino-2-{[2-(1-piperazinyl)ethyl]amino}ethyl)phosphonic acid | C8 H21 N4 O3 P | 13.958 | 5193700.974 | 252.13518 | 251.1279 |
|  | 3'-Amino-2',3'-dideoxyadenosine | C10 H14 N6 O2 | 13.283 | 5080467.19 | 250.11712 | 249.10985 |
|  | (5R)-9-Formyl-13-isopropyl-5-methyl-2-(2-{2-[4-(methylsulfanyl)phenyl]acetoxy}ethyl)tetracyclo[7.4.0.0~2,11~.0~4,8~]tridec-12-ene-1-carboxylic acid | C30 H38 O5 S | 12.099 | 4891528.872 | 510.24473 | 509.23746 |
|  | 4-{[(4-Chlorophenyl)sulfanyl]methyl}-6-(2-methoxyphenyl)-2,2-dimethyl-1,2-dihydroquinoline | C25 H24 Cl N O S | 12.451 | 4517182.774 | 421.12797 | 420.1207 |
|  | 3-Methyl-3-(3-{2-methyl-2-[3-(2-methyl-2-{3-[2-(2-methyl-1,3-dioxolan-2-yl)-2-propanyl]-1,2-oxazol-5-yl}propyl)-1,2-oxazol-5-yl]propyl}-1,2-oxazol-5-yl)butanenitrile | C29 H40 N4 O5 | 10.198 | 4395829.506 | 524.29738 | 523.2901 |
|  | 1-(Butylsulfanyl)borepane | C10 H21 B S | 13.586 | 4230147.554 | 184.1449 | 183.13762 |
|  | [Similar to: 4,5-Dicaffeoylquinic acid; ΔMass: 0.1570 Da] | C21 H46 Cl N4 O6 P | 10.103 | 4062689.871 | 516.28377 | 515.27649 |
|  | 3-(3-Furoylamino)-L-alanyl-L-valyl-N-{(2S,3R)-4-[(3-carboxyphenyl)amino]-3-hydroxy-4-oxo-1-phenyl-2-butanyl}-L-leucinamide | C36 H46 N6 O9 | 18.328 | 3845081.864 | 706.33339 | 705.32611 |
|  | L-Leucyl-L-prolyl-L-lysyl-L-threonyl-L-alanine | C24 H44 N6 O7 | 8.255 | 3406330.992 | 528.32881 | 527.32153 |

Table 6: LCMS of hexane extract from *Ajuga integrifolia*

| **S.NO** | **Name** | **Formula** | **RT (Min)** | **Area(max.)** | **Cal MW.** | **m/z** |
| --- | --- | --- | --- | --- | --- | --- |
|  | A-12(13)-EpODE | C18 H30 O3 | 18.882 | 1291506758 | 294.21852 | 293.21127 |
|  | 13(S)-HOTrE | C18 H30 O3 | 19.042 | 718816700.6 | 294.21846 | 293.21121 |
|  | NP-020521 | C18 H32 O3 | 20.042 | 598092764.5 | 296.234 | 295.22675 |
|  | 16-Hydroxyhexadecanoic acid | C16 H32 O3 | 24.092 | 503649757.2 | 272.23414 | 271.22687 |
|  | Tetrakis(2-oxiranylmethyl) 3,3',3'',3'''-(1,1,3,3-cyclohexanetetrayl)tetrapropanoate | C30 H44 O12 | 16.45 | 395021437.7 | 596.2812 | 595.27393 |
|  | Pinolenic acid | C18 H30 O2 | 24.001 | 363217127.2 | 278.22349 | 277.21622 |
|  | L-Tyrosyl-L-prolyl-N~5~-(diaminomethylene)-L-ornithine | C20 H30 N6 O5 | 14.184 | 296430902 | 434.2287 | 479.22681 |
|  | 2-Methyl-2-propanyl 4-[(3R)-3-{[(benzyloxy)carbonyl]amino}-4-methoxy-4-oxobutyl]-1-piperidinecarboxylate | C23 H34 N2 O6 | 21.713 | 269415108.2 | 434.24171 | 433.23444 |
|  | 9(Z),11(E)-Conjugated linoleic acid | C18 H32 O2 | 25.528 | 168975347.4 | 280.23912 | 279.23184 |
|  | Ethyl N~2~-(3,4-dimethoxybenzyl)-N~5~-{[(2-methyl-2-propanyl)oxy]carbonyl}-L-ornithinate | C21 H34 N2 O6 | 24.03 | 146226697.2 | 410.24162 | 409.23434 |
|  | 13(S)-HOTrE | C18 H30 O3 | 19.813 | 143277123.8 | 294.2185 | 329.1879 |
|  | Embelin | C17 H26 O4 | 15.635 | 127353371.7 | 294.18228 | 293.17501 |
|  | [Similar to: NP-003553; ΔMass: 93.9804 Da] | C17 H33 N6 O5 P | 20.305 | 126243861.7 | 432.22612 | 431.21884 |
|  | (4E)-7-(Chlorocarbonyl)-2-pentyl-4-dodecenoic acid | C18 H31 Cl O3 | 20.058 | 122110163 | 330.19494 | 329.18771 |
|  | Palmitic acid | C16 H32 O2 | 27.234 | 118701134.2 | 256.23908 | 255.23178 |
|  | MFCD03672170 | C17 H26 N6 O3 | 18.879 | 110939186 | 362.20552 | 361.19824 |
|  | MFCD00059633 | C14 H28 O3 | 20.521 | 82548711.52 | 244.20283 | 243.19556 |
|  | 13(S)-HpOTrE | C18 H30 O4 | 16.772 | 76950506.83 | 310.21347 | 309.2062 |
|  | Glycol stearate | C20 H40 O3 | 26.933 | 73403681.07 | 328.29677 | 327.28949 |
|  | Juniperic acid | C16 H32 O3 | 23.077 | 70907478.9 | 272.23439 | 271.22711 |
|  | N-[(2S)-2-Butanyl]-N~2~-[2-ethyl-2-(isobutyrylamino)butanoyl]-L-argininamide | C20 H40 N6 O3 | 22.174 | 69490393.35 | 412.31749 | 411.31021 |
|  | Oleoyl-L-α-lysophosphatidic acid | C21 H41 O7 P | 24.032 | 68727047.33 | 436.25728 | 435.25 |
|  | MFCD03672170 | C17 H26 N6 O3 | 19.042 | 67699847.14 | 362.20546 | 361.19818 |
|  | (4E)-7-(Chlorocarbonyl)-2-pentyl-4-dodecenoic acid | C18 H31 Cl O3 | 20.154 | 65234761.72 | 330.19501 | 329.18781 |
|  | 7-(2-Ethoxyethyl)-1,3-dimethyl-8-[(4-methyl-1-piperazinyl)methyl]-3,7-dihydro-1H-purine-2,6-dione | C17 H28 N6 O3 | 20.039 | 53717113.25 | 364.2212 | 363.21393 |
|  | (±)9-HpODE | C18 H32 O4 | 17.952 | 51484757.48 | 312.22929 | 311.22202 |
|  | NP-020521 | C18 H32 O3 | 22.955 | 48288483.02 | 296.23414 | 295.22687 |
|  | [Similar to: NP-001134; ΔMass: 46.0035 Da] | C13 H20 N10 O2 | 21.168 | 47830994.89 | 348.17775 | 347.17047 |
|  | 15-Hydroxypentadecanoic acid | C15 H30 O3 | 21.541 | 44831967.37 | 258.21849 | 257.21121 |
|  | Tetrakis(2-methyl-2-butanyl) 1,2,4,5-benzenetetracarboperoxoate | C30 H46 O12 | 15.2 | 44749828.86 | 598.29677 | 597.28949 |
|  | {4-[(Cyclopropylmethyl)sulfinyl]phenyl}boronic acid | C10 H13 B O3 S | 7.411 | 44660511.94 | 224.06732 | 223.06004 |
|  | Tetradecyl 4-chloro-4-oxobutanoate | C18 H33 Cl O3 | 21.033 | 44557237.37 | 332.2108 | 331.20355 |
|  | 13(S)-HpOTrE | C18 H30 O4 | 16.954 | 44435036.91 | 310.21348 | 309.20621 |
|  | 4-(4,5-Diphenyl-1H-imidazol-2-yl)benzohydrazide | C22 H18 N4 O | 26.081 | 41992549.9 | 354.14857 | 353.1413 |
|  | 7-(2-Ethoxyethyl)-1,3-dimethyl-8-[(4-methyl-1-piperazinyl)methyl]-3,7-dihydro-1H-purine-2,6-dione | C17 H28 N6 O3 | 20.17 | 40448508.29 | 364.2213 | 363.21402 |
|  | N-[(2R)-2-{4-[4-(2-Methyl-1H-imidazol-1-yl)butyl]phenyl}propanoyl]-L-seryl-N-(2-cyclohexylethyl)-L-lysinamide | C34 H54 N6 O4 | 18.886 | 39816079.24 | 610.41847 | 609.41119 |
|  | (+/-)9-HpODE | C18 H32 O4 | 16.358 | 38750706.42 | 312.22913 | 311.22185 |
|  | myristyl lactate | C17 H34 O3 | 24.077 | 38019220.51 | 286.24971 | 285.24243 |
|  | Diethyl (1,6-hexanediyldi-6,8-phenanthridinediyl)biscarbamate | C38 H38 N4 O4 | 13.587 | 37357495.02 | 614.29152 | 613.28424 |
|  | N-[(2S)-2-Butanyl]-N~2~-[2-ethyl-2-(isobutyrylamino)butanoyl]-L-argininamide | C20 H40 N6 O3 | 21.868 | 33741996.1 | 412.31754 | 411.31027 |
|  | 12-HSA | C18 H36 O3 | 25.633 | 32724973.27 | 300.26533 | 299.25806 |
|  | (±)9-HpODE | C18 H32 O4 | 20.727 | 31303231.17 | 312.22917 | 293.21136 |
|  | Diethyl diallylmalonate | C13 H20 O4 | 16.124 | 30153744.89 | 240.13511 | 239.12784 |
|  | DECYL GALLATE | C17 H26 O5 | 16.316 | 28824176.09 | 310.17711 | 309.16983 |
|  | 2,6-Dichlorotoluene | C7 H6 Cl2 | 0.582 | 28436129.34 | 159.98437 | 158.9771 |
|  | 1,4-Diethyl-3,6-bis(4-morpholinylmethyl)tetrahydroimidazo[4,5-d]imidazole-2,5(1H,3H)-dione | C18 H32 N6 O4 | 21.495 | 26574720.2 | 396.24974 | 395.24255 |
|  | Tetradecyl 4-chloro-4-oxobutanoate | C18 H33 Cl O3 | 21.371 | 26271213.8 | 332.21077 | 331.20349 |
|  | (±)12(13)-DiHOME | C18 H34 O4 | 17.327 | 26064986.73 | 314.24486 | 313.23758 |
|  | Mitoxantrone | C22 H28 N4 O6 | 16.829 | 24933485.44 | 444.20311 | 489.20129 |
|  | 13(S)-HpOTrE | C18 H30 O4 | 15.985 | 24066976.46 | 310.21342 | 309.20615 |
|  | [Similar to: 4-Dodecylbenzenesulfonic acid; ΔMass: 68.0924 Da] | C17 H39 Cl N6 O2 | 28.23 | 23867203.09 | 394.28392 | 393.27664 |
|  | 12-HSA | C18 H36 O3 | 27.228 | 23355620.44 | 300.26527 | 299.258 |
|  | Cannabidiolic acid | C22 H30 O4 | 25.187 | 22701765.57 | 358.21321 | 357.20593 |
|  | NP-011548 | C18 H34 O3 | 24.758 | 22667640.75 | 298.24977 | 297.24249 |
|  | (±)9-HpODE | C18 H32 O4 | 17.566 | 22453427.8 | 312.22908 | 311.2218 |
|  | 13(S)-HOTrE | C18 H30 O3 | 18.02 | 22177202.77 | 294.21867 | 293.2114 |
|  | (E)-parinaric acid | C18 H28 O2 | 18.82 | 22175352.85 | 276.20775 | 275.20047 |
|  | N-[(1S)-5-Amino-1-{5-[(1S)-1-amino-2-hydroxyethyl]-1,3,4-oxadiazol-2-yl}pentyl]-4-piperidinecarboxamide | C15 H28 N6 O3 | 24.139 | 21300114.1 | 340.2212 | 339.21393 |
|  | Oleic acid | C18 H34 O2 | 27.618 | 20851916.57 | 282.25477 | 281.2475 |
|  | N~2~-Acetyl-L-lysyl-N-(6-aminohexyl)-L-lysinamide | C20 H42 N6 O3 | 23.03 | 20374660.52 | 414.33308 | 413.32581 |
|  | A-12(13)-EpODE | C18 H30 O3 | 18.669 | 20372602.38 | 294.21861 | 293.21133 |
|  | MFCD00083370 | C22 H44 O3 | 26.247 | 20191048.98 | 356.32786 | 355.32059 |
|  | MFCD00152308 | C24 H44 O4 | 24.027 | 19546310.99 | 396.32231 | 395.31503 |
|  | A-12(13)-EpODE | C18 H30 O3 | 18.286 | 18556447.97 | 294.21864 | 293.21136 |
|  | (+/-)9-HpODE | C18 H32 O4 | 17.019 | 17883452.74 | 312.22905 | 311.22177 |
|  | 4,9-Bis{[2-(4-methyl-1-piperazinyl)ethyl]amino}-2,7-bis[2-(4-morpholinyl)ethyl]benzo[lmn][3,8]phenanthroline-1,3,6,8(2H,7H)-tetrone | C40 H58 N10 O6 | 23.168 | 16661533.78 | 774.45302 | 773.44574 |
|  | N-[(2S)-2-Butanyl]-N~2~-[2-ethyl-2-(isobutyrylamino)butanoyl]-L-argininamide | C20 H40 N6 O3 | 21.095 | 16567880.22 | 412.31758 | 411.3103 |
|  | 9,10-Dihydroxystearic acid | C18 H36 O4 | 19.262 | 16480231.29 | 316.26027 | 315.25299 |
|  | (3S)-3-{[(3S)-3-({[(1R,2R)-2-{[(3R)-3-{[(3R)-3-({[(1R,2R)-2-Aminocyclohexyl]carbonyl}amino)-4-hydroxybutanoyl]amino}-4-methylpentanoyl]amino}cyclohexyl]carbonyl}amino)-5-methylhexanoyl]amino}hexanedio ic acid | C37 H64 N6 O10 | 29.882 | 16323632 | 752.46834 | 751.46106 |
|  | Corchorifatty acid F | C18 H32 O5 | 13.698 | 15964508.46 | 328.22374 | 327.21646 |
|  | 1,4-Diethyl-3,6-bis(4-morpholinylmethyl)tetrahydroimidazo[4,5-d]imidazole-2,5(1H,3H)-dione | C18 H32 N6 O4 | 21.633 | 15894380.2 | 396.24979 | 395.24258 |
|  | 9,10-Dihydroxystearic acid | C18 H36 O4 | 18.47 | 14831948.79 | 316.26036 | 315.25308 |
|  | Tretinoin | C20 H28 O2 | 19.813 | 14386497.48 | 300.20787 | 299.20059 |
|  | N-[(2S)-2-Butanyl]-N~2~-[2-ethyl-2-(isobutyrylamino)butanoyl]-L-argininamide | C20 H40 N6 O3 | 21.645 | 13967444.16 | 412.31767 | 411.31039 |
|  | Di-n-Amyl phthalate | C18 H26 O4 | 16.319 | 13839949.13 | 306.18217 | 305.1749 |
|  | 1-(2-NITROPHENOXY)OCTANE | C14 H21 N O3 | 21.773 | 13678209.9 | 251.15109 | 250.14381 |
|  | N-Methyl-N-{4-[(E)-2-phenylvinyl]phenyl}-1,3,2-oxazaphosphinan-2-amine 2-oxide | C18 H21 N2 O2 P | 24.449 | 13628162.41 | 328.13322 | 327.12595 |
|  | Cyclo(D-alanyl-D-prolyl-D-alanyl-L-leucyl-D-alpha-aspartylglycyl) | C23 H36 N6 O8 | 14.178 | 13585250.5 | 524.26027 | 523.25299 |
|  | JWH-398 | C24 H22 Cl N O | 27.298 | 13528207.26 | 375.13811 | 374.13083 |
|  | 15-Hydroxypentadecanoic acid | C15 H30 O3 | 22.85 | 13322698.6 | 258.21861 | 257.21133 |
|  | (+/-)13-HODE | C18 H32 O3 | 22.089 | 12931926.14 | 296.23408 | 295.22681 |
|  | 10-CHLORO-9-HYDROXYSTEARIC ACID | C18 H35 Cl O3 | 22.605 | 12787380.19 | 334.22651 | 333.21924 |
|  | ricinelaidic acid | C18 H34 O3 | 20.664 | 12434506.03 | 298.24974 | 297.24246 |
|  | N-[(2R)-6-Amino-1-(hydroxyamino)-1-oxo-2-hexanyl]-10-(2-{[(diaminomethylene)amino]methyl}phenyl)decanamide | C24 H42 N6 O3 | 23.546 | 12176861.39 | 462.33314 | 461.32587 |
|  | 13(S)-HpOTrE | C18 H30 O4 | 18.971 | 12033576.95 | 310.21342 | 309.20615 |
|  | N-{4-[1-Benzyl-5-(methoxymethyl)-7-methyl-4-oxo-1,2,3,4-tetrahydropyrido[2,3-d]pyrimidin-2-yl]phenyl}acetamide | C25 H26 N4 O3 | 25.21 | 12000649.27 | 430.20057 | 429.1933 |
|  | NP-005013 | C16 H12 O5 | 15.003 | 11892693.42 | 284.06755 | 283.06027 |
|  | N-[(2S)-2-Butanyl]-N~2~-[2-ethyl-2-(isobutyrylamino)butanoyl]-L-argininamide | C20 H40 N6 O3 | 20.906 | 11516265.83 | 412.31758 | 411.3103 |
|  | 13(S)-HpOTrE | C18 H30 O4 | 19.482 | 11421439.81 | 310.21351 | 309.20624 |
|  | Diethyl (3-{[(11aS)-7-methoxy-5-oxo-2,3,5,11a-tetrahydro-1H-pyrrolo[2,1-c][1,4]benzodiazepin-8-yl]oxy}propyl)phosphonate | C20 H29 N2 O6 P | 18.855 | 11328107.17 | 424.17579 | 423.16852 |
|  | {4-[(Diaminomethylene)amino]-1-azabicyclo[3.1.0]hex-2-yl}(valylamino)acetic acid | C13 H24 N6 O3 | 20.52 | 11134676.15 | 312.19017 | 311.18289 |
|  | DOA | C22 H42 O4 | 24.952 | 10869389.39 | 370.30693 | 369.29965 |
|  | N~2~-(Diisopropoxyphosphoryl)-N-{4-[(E)-2-phenylvinyl]phenyl}-L-valinamide | C25 H35 N2 O4 P | 28.23 | 10851145.23 | 458.23225 | 457.22498 |
|  | (6R,7S)-7-{[(2E)-2-(2-Amino-1,3-thiazol-4-yl)-2-(methoxyimino)acetyl]amino}-3-(methylsulfonyl)-8-oxo-1-azabicyclo[4.2.0]oct-2-ene-2-carboxylic acid | C15 H17 N5 O7 S2 | 23.973 | 10794339.84 | 443.05827 | 442.05099 |
|  | N-[5-(Imidazo[1,2-a]pyrimidin-2-yl)-2-methylphenyl]-2-phenylbutanamide | C23 H22 N4 O | 28.49 | 10085578.37 | 370.17976 | 369.17249 |
|  | Protirelin | C16 H22 N6 O4 | 15.636 | 10074249.79 | 362.16939 | 361.16211 |
|  | [Similar to: 4-Dodecylbenzenesulfonic acid; ΔMass: 68.0923 Da] | C17 H39 Cl N6 O2 | 27.743 | 9849642 | 394.28383 | 393.27655 |
|  | (2R)-N-(2-{[(2S)-5-Carbamimidamido-1-oxo-2-pentanyl]amino}-2-oxoethyl)-N-isobutyl-2-piperidinecarboxamide | C18 H34 N6 O3 | 20.779 | 9731608.248 | 382.2707 | 381.26343 |
|  | Dodecylsuccinic Anhydride | C16 H28 O3 | 23.988 | 9676686.151 | 268.20277 | 267.1955 |
|  | 13(S)-HOTrE | C18 H30 O3 | 18.495 | 9429710.663 | 294.21861 | 293.21133 |
|  | NP-006255 | C17 H26 O4 | 21.495 | 9257506.702 | 294.18226 | 293.17499 |
|  | N,N-Bis(2-hydroxyethyl)dodecanamide | C16 H33 N O3 | 16.168 | 8943063.576 | 287.24507 | 286.23779 |
|  | NP-016067 | C32 H50 O4 | 26.094 | 8880145.248 | 498.36913 | 497.36188 |
|  | N-[3-(Dimethylamino)-2,2-dimethylpropyl]-N~3~-{2-hexyl-7-[(1E)-3-(hydroxyamino)-3-oxo-1-propen-1-yl]imidazo[1,2-a]pyridin-3-yl}-beta-alaninamide | C26 H42 N6 O3 | 25.726 | 8831537.152 | 486.33296 | 485.32568 |
|  | (5R)-2,4-Dideoxy-3-O-(2,6-dideoxy-alpha-D-arabino-hexopyranosyl)-1-C-{(2S,3R,4S)-3-hydroxy-4-[(2R,3S,4Z,9S,10S,11R)-10-hydroxy-3,15-dimethoxy-7,9,11,13-tetramethyl-16-oxooxacyclohexadeca-4,6,12,14-tet raen-2-yl]-2-pentanyl}-4-methyl-5-[(1E,3E)-1,3-pentadien-1-yl]-alpha-D-threo-pentopyranose | C43 H68 O12 | 24.199 | 8672765.316 | 776.46821 | 775.46094 |
|  | 5-OxoETE | C20 H30 O3 | 22.375 | 8631789.216 | 318.2184 | 317.21112 |
|  | Pentosidine | C17 H26 N6 O4 | 16.741 | 8610914.88 | 378.2007 | 377.19342 |
|  | 11-(2-Sulfophenyl)undecanoic acid | C17 H26 O5 S | 25.765 | 8406875.004 | 342.14863 | 341.14136 |
|  | (2E)-3-(4-Chlorophenyl)-N-[(5alpha)-17-(cyclopropylmethyl)-6-oxo-3-(2-propyn-1-yloxy)-4,5-epoxymorphinan-14-yl]acrylamide | C32 H31 Cl N2 O4 | 19.386 | 8110796.954 | 542.19545 | 541.18817 |
|  | NP-006255 | C17 H26 O4 | 18.642 | 7993647.621 | 294.18236 | 293.17508 |
|  | 1-[6-(4-Imino-2-oxo-1,3,5-triazacyclotridecan-1-yl)hexyl]-3-(2-propyn-1-yl)guanidine | C20 H37 N7 O | 24.06 | 7937056.208 | 391.30717 | 390.2999 |
|  | 13(S)-HOTrE | C18 H30 O3 | 20.394 | 7906007.354 | 294.21864 | 293.21136 |
|  | N-[(2S)-2-Butanyl]-N~2~-[2-ethyl-2-(isobutyrylamino)butanoyl]-L-argininamide | C20 H40 N6 O3 | 20.004 | 7893958.511 | 412.31737 | 411.31009 |
|  | NP-020521 | C18 H32 O3 | 19.41 | 7707756.903 | 296.23405 | 295.22678 |
|  | N~2~-Acetyl-L-lysyl-N-(6-aminohexyl)-L-lysinamide | C20 H42 N6 O3 | 21.595 | 7705584.615 | 414.33314 | 413.32587 |
|  | (2R)-N-(2-{[(2S)-5-Carbamimidamido-1-oxo-2-pentanyl]amino}-2-oxoethyl)-N-isobutyl-2-piperidinecarboxamide | C18 H34 N6 O3 | 20.451 | 7589677.466 | 382.27073 | 381.26346 |
|  | ricinelaidic acid | C18 H34 O3 | 21.285 | 7565400.493 | 298.24974 | 297.24246 |
|  | myristyl lactate | C17 H34 O3 | 23.966 | 7370210.779 | 286.24977 | 285.24249 |
|  | N-[(1S)-5-Amino-1-{5-[(1S)-1-amino-2-hydroxyethyl]-1,3,4-oxadiazol-2-yl}pentyl]-4-piperidinecarboxamide | C15 H28 N6 O3 | 23.079 | 7264159.48 | 340.22139 | 339.21411 |
|  | N-[(2S)-2-Butanyl]-N~2~-[2-ethyl-2-(isobutyrylamino)butanoyl]-L-argininamide | C20 H40 N6 O3 | 22.423 | 7227137.193 | 412.31749 | 411.31021 |
|  | 6-({2-[(6-tert-Butyl-4,5,6,7-tetrahydro-1-benzothiophen-3-yl)carbonyl]hydrazino}carbonyl)-3,4-dimethylcyclohex-3-ene-1-carboxylic acid | C23 H32 N2 O4 S | 20.029 | 6918102.255 | 432.2082 | 431.20093 |
|  | arabidopside A | C43 H66 O12 | 23.023 | 6879402.071 | 774.45308 | 773.4458 |
|  | Methyl 12-hydroxystearate | C19 H38 O3 | 25.31 | 6847753.817 | 314.28114 | 313.27386 |
|  | 7-Benzyl-8-[4-(2-hydroxyethyl)-1-piperazinyl]-3-methyl-3,7-dihydro-1H-purine-2,6-dione | C19 H24 N6 O3 | 18.556 | 6846892.555 | 384.19011 | 383.18283 |
|  | Artemotil | C17 H28 O5 | 15.444 | 6588945.319 | 312.19273 | 311.18546 |
|  | Mono(2-ethylhexyl) phthalate (MEHP) | C16 H22 O4 | 19.285 | 6342356.074 | 278.1508 | 277.14352 |
|  | Juniperic acid | C16 H32 O3 | 22.807 | 6276099.837 | 272.23436 | 271.22708 |
|  | TOFA | C19 H32 O4 | 19.305 | 6150751.82 | 324.22905 | 323.22177 |
|  | 13(S)-HpOTrE | C18 H30 O4 | 18.497 | 5986665.778 | 310.21351 | 309.20624 |
|  | Methyl (5xi,18alpha)-3,12-dioxoolean-9(11)-en-28-oate | C31 H46 O4 | 27.353 | 5948776.642 | 482.33775 | 481.33047 |
|  | 2,4,6,8-Decatetraenedioic Acid Mono[4-(1,2-epoxy-1,5-dimethyl-4-hexenyl)-5-methoxy-1-oxaspiro[2.5]oct-6-yl] Ester | C26 H34 O7 | 28.843 | 5918502.841 | 458.23207 | 457.22479 |
|  | DECYL GALLATE | C17 H26 O5 | 15.061 | 5633370.481 | 310.17714 | 309.16986 |
|  | (3S)-1-[(4-Methylphenyl)sulfonyl]-3-pyrrolidinyl 4-methylbenzenesulfonate | C18 H21 N O5 S2 | 20.328 | 5262414.038 | 395.08522 | 394.07794 |
|  | 15-Hydroxypentadecanoic acid | C15 H30 O3 | 21.405 | 5259225.376 | 258.21852 | 257.21124 |
|  | [Similar to: 4-Dodecylbenzenesulfonic acid; ΔMass: 0.0143 Da] | C14 H26 N6 O3 | 21.543 | 5222392.576 | 326.20585 | 325.19858 |
|  | 2'-C-Methyl-5'-O-[phenoxy({2-[(2-propylpentanoyl)oxy]ethyl}amino)phosphoryl]cytidine | C26 H39 N4 O9 P | 20.548 | 4979617.918 | 582.24592 | 581.23865 |
|  | Glycol stearate | C20 H40 O3 | 24.023 | 4930788.115 | 328.29658 | 327.28931 |
|  | Methyl phaeophorbide | C36 H38 N4 O5 | 26.19 | 4906382.676 | 606.28218 | 605.2749 |
|  | 15-Keto prostaglandin A1 | C20 H30 O4 | 16.425 | 4654900.069 | 334.21333 | 333.20605 |
